# Pharmacological and genetic reappraisals of protease and oxidative stress pathways in a mouse model of obstructive lung diseases

**DOI:** 10.1038/srep39305

**Published:** 2016-12-16

**Authors:** Tsuyoshi Shuto, Shunsuke Kamei, Hirofumi Nohara, Haruka Fujikawa, Yukihiro Tasaki, Takuya Sugahara, Tomomi Ono, Chizuru Matsumoto, Yuki Sakaguchi, Kasumi Maruta, Ryunosuke Nakashima, Taisei Kawakami, Mary Ann Suico, Yoshitaka Kondo, Akihito Ishigami, Toru Takeo, Ken-ichiro Tanaka, Hiroshi Watanabe, Naomi Nakagata, Kohei Uchimura, Kenichiro Kitamura, Jian-Dong Li, Hirofumi Kai

**Affiliations:** 1 Department of Molecular Medicine, Graduate School of Pharmaceutical Sciences, Kumamoto University, 5-1 Oe-Honmachi, Chuo-ku, Kumamoto 862-0973, Japan; 2 Program for Leading Graduate Schools “HIGO (Health life science: Interdisciplinary and Glocal Oriented) Program”, Kumamoto University, 5-1 Oe-Honmachi, Chuo-ku, Kumamoto 862-0973, Japan; 3 Molecular Regulation of Aging, Tokyo Metropolitan Institute of Gerontology, 35-2 Sakae-cho, Itabashi-ku, Tokyo 173-0015, Japan; 4 Division of Reproductive Engineering, Center for Animal Resources and Development (CARD), Kumamoto University, 2-2-1 Honjo, Chuo-ku, Kumamoto, 860–0811, Japan; 5 Laboratory of Bio-Analytical Chemistry, Research Institute of Pharmaceutical Sciences, Musashino University, 1-1-20 Shinmachi, Nishitokyo-shi, Tokyo 202-8585, Japan; 6 Department of Biopharmaceutics, Graduate School of Pharmaceutical Sciences, Kumamoto University, 5-1 Oe-honmachi, Chuo-ku, Kumamoto 862-0973, Japan; 7 University of Yamanashi School of Medicine, 1110 Shimokato, Chuo, Yamanashi 409-3898, Japan; 8 Center for Inflammation, Immunity & Infection, Institute for Biomedical Sciences, Georgia State University, 714 Petit Science Center, 100 Piedmont Ave SE, Atlanta, GA30303, USA

## Abstract

Protease-antiprotease imbalance and oxidative stress are considered to be major pathophysiological hallmarks of severe obstructive lung diseases including chronic obstructive pulmonary disease (COPD) and cystic fibrosis (CF), but limited information is available on their direct roles in the regulation of pulmonary phenotypes. Here, we utilized βENaC-transgenic (Tg) mice, the previously established mouse model of severe obstructive lung diseases, to produce lower-mortality but pathophysiologically highly useful mouse model by backcrossing the original line with C57/BL6J mice. C57/BL6J-βENaC-Tg mice showed higher survival rates and key pulmonary abnormalities of COPD/CF, including mucous hypersecretion, inflammatory and emphysematous phenotypes and pulmonary dysfunction. DNA microarray analysis confirmed that protease- and oxidative stress-dependent pathways are activated in the lung tissue of C57/BL6J-βENaC-Tg mice. Treatments of C57/BL6J-βENaC-Tg mice with a serine protease inhibitor ONO-3403, a derivative of camostat methylate (CM), but not CM, and with an anti-oxidant N-acetylcystein significantly improved pulmonary emphysema and dysfunction. Moreover, depletion of a murine endogenous antioxidant vitamin C (VC), by genetic disruption of VC-synthesizing enzyme SMP30 in C57/BL6J-βENaC-Tg mice, exaggerated pulmonary phenotypes. Thus, these assessments clarified that protease-antiprotease imbalance and oxidative stress are critical pathways that exacerbate the pulmonary phenotypes of C57/BL6J-βENaC-Tg mice, consistent with the characteristics of human COPD/CF.

Pulmonary emphysema and dysfunction are pathophysiological characteristics of severe obstructive lung diseases including chronic obstructive pulmonary disease (COPD) and cystic fibrosis (CF). In these disorders, defective mucus clearance, excessive inflammation, protease-antiprotease imbalance and oxidative stress have been considered to influence the seriousness^1^,^2^,^3^,^4^. Because COPD is a worldwide leading cause of morbidity and mortality^1^ and CF is the most common lethal inherited disorder in Caucasians^3^, identification of the key molecules and pathways underlying disease pathogenesis has been the subject of extensive research for many years. Experimentally, ideal murine model that exhibits critical pulmonary phenotypes of COPD/CF, such as mucus obstruction, goblet cell metaplasia, neutrophilic inflammation and poor bacterial clearance, has been uniquely established by inducing airway-specific overexpression of the epithelial Na^+^ channel β subunit in mice (βENaC-Tg mice)^5^. Importantly, the same group further revealed by histological and morphological analysis that βENaC-Tg mice exhibit not only emphysematous phenotype but also pulmonary dysfunction, and these pulmonary abnormalities were strongly associated with those typically observed in patients with COPD and CF^6^,^7^.

ENaC is a sodium ion channel that is expressed in the apical membrane of polarized epithelial cells particularly in the lung, the kidney (primarily in the collecting tubules) and the colon^8^,^9^. Over-activation of ENaC by airway-targeted βENaC overexpression leads to the generation of concentration gradient of sodium ions (e.g., sodium ions going from outside to inside of the cell) followed by over-absorption of water into the cells, which results in dysregulated airway mucus production and airway clearance^5^,^9^. Based on the lines of evidence showing that the expression and function of ENaC were inversely associated with lung function in CF patients^9^,^10^ and could be augmented in COPD patients^11^,^12^, βENaC-Tg mice could be valuable tools for exploring mucus obstructive phenotypes of COPD and CF *in vivo*. However, the mortality of βENaC-Tg mice with a mixed C3H/HeN:C57/BL6N background was extremely high^5^, which limits usage of the original βENaC-Tg line as an animal model of acute and fulminant airway diseases. Importantly, Livraghi-Butrico, *et al*., showed that the genetic background of βENaC-Tg mice strongly affects survival and disease severity in the mice^13^. Because Johannesson, *et al*., also clearly demonstrated that C57/BL6 back-crossing has improved survival, the βENaC-Tg mice with C57/BL6 background could be considered as an ideal animal model that represents the phenotypes of human obstructive airway diseases^14^. However, despite the increasing evidence of their usefulness in COPD and CF researches, the significance of the C57/BL6-βENaC-Tg mice has been demonstrated by only a few groups worldwide, suggesting that comprehensive analysis to determine the specific molecules and pathways that could contribute to the pathogenesis of C57/BL6-βENaC-Tg mice are further needed.

In the present study, we back-crossed high-mortality βENaC-Tg mice with C57/BL6J line and improved the survival of mice. In addition to the typical pulmonary phenotypes of COPD and CF in the C57/BL6J line, proteases and oxidative stress pathways, critical signals that are normally activated in the lung tissues of COPD and CF patients, were also strongly activated in the lung tissue of C57/BL6J-βENaC-Tg mice. Consistently, pharmacological approaches by inhibiting endogenous proteases and oxidative stress pathways and genetic approach that eliminates endogenous anti-oxidant vitamin C (VC) confirmed the indispensable role of these pathways in the pathogenesis of βENaC-Tg mice with C57/BL6J background. Overall, our study emphasizes protease-antiprotease imbalance and oxidative stress as exacerbating factors in the pathogenesis of C57/BL6J-βENaC-Tg mice as it is normally observed in the patients with COPD and CF.

## Results

### Characterization of mucus obstructive and inflammatory phenotypes in the pulmonary tissue of βENaC-Tg mice with C57/BL6J background

**Figure 1:**
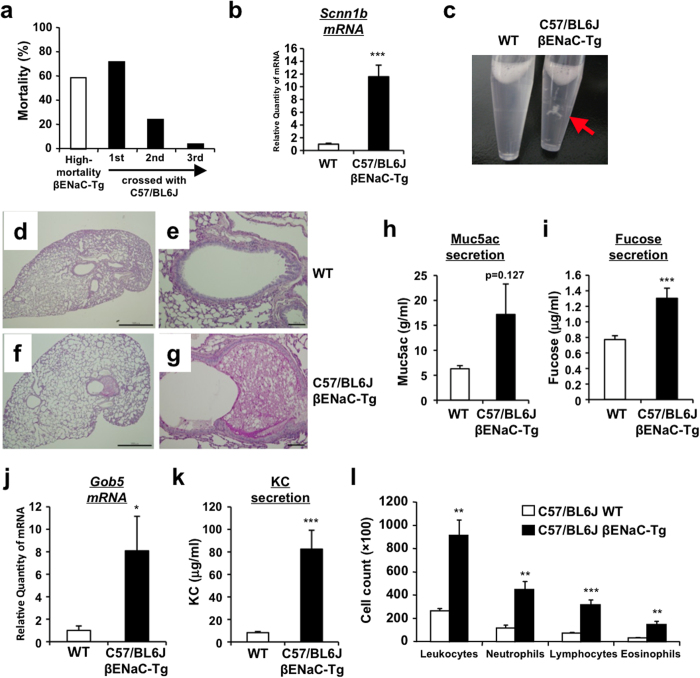
Airway mucus obstruction and inflammation of low-mortality airway-specific βENaC-Tg mice with C57/BL6J background. (**a**) Mortality of βENaC-Tg mice backcrossed with C57/BL6J mice for three generations. (**b**) Pulmonary mRNA expression of *Scnn1b* (βENaC) gene in WT (*n* = 14) and C57/BL6J-βENaC-Tg (*n* = 17) mice quantified by Q-RT-PCR. (**c**) Mucus plug in BALF of WT and C57/BL6J-βENaC-Tg mice. (**d**–**g**) Representative data (*n* = 4–5) of PAS and alcian blue-stained section. Mucus plug observed in C57/BL6J-βENaC-Tg mice (**f,g**), but not in WT mice (**d**,**e**). Scale bars, 1,000 μm (**d**,**f**) and 100 μm (**e**,**g**). (**h**–**k**) Muc5ac (**h**), fucose (**i**) and KC (**k**) concentration in BALF of WT (*n* = 9) and C57/BL6J-βENaC-Tg WT (*n* = 11) mice. Pulmonary Gob5 gene expression (**j**) in WT (*n* = 14) and C57/BL6J-βENaC-Tg (*n* = 17) mice. (**l**) Inflammatory cell count in BALF of WT (*n* = 4) and C57/BL6J-βENaC-Tg WT (*n* = 5) mice. ^*^p < 0.05, ^**^p < 0.01, ^***^p < 0.001, versus WT; Student’s *t* test.

To confirm the pulmonary phenotypes of βENaC-Tg mice with C57/BL6J background, we first back-crossed the high-mortality βENaC-Tg mice (JAX#006438) with C57/BL6J line. Early mortality was reduced from 59% to 3% during three generations (Fig. 1a). Consistent with a higher expression of Scnn1b (βENaC) gene in lung tissue of C57/BL6J-βENaC-Tg mice (Fig. 1b), typical COPD/CF-like phenotypes were clearly observed in C57/BL6J-βENaC-Tg mice, such as the presence of mucus plug and obstruction (Fig. 1c–g), increased expression of Muc5ac and fucose levels in bronchoalveolar lavage fluid (BALF) (Fig. 1h,i), gob5 gene induction in lung tissue (Fig. 1j) and immune cells-associated pulmonary inflammation (Fig. 1k,l).

### Evaluation of emphysematous phenotype, pulmonary mechanics and function in C57/BL6J-βENaC-Tg mice

**Figure 2:**
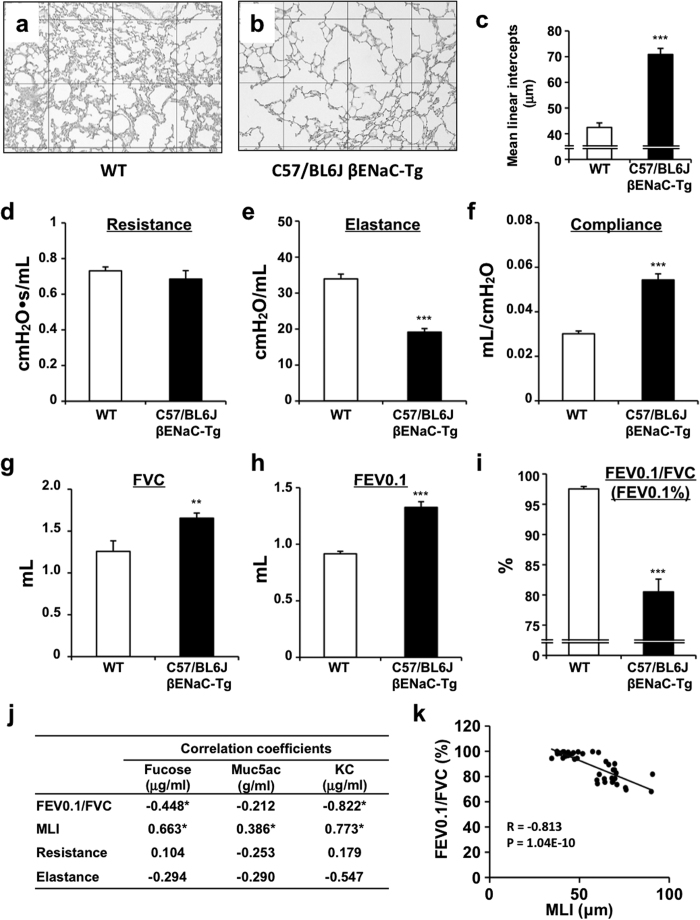
Pulmonary emphysema and dysfunction C57/BL6J-βENaC-Tg mice. (**a**–**c**) Emphysematous phenotypes of WT and C57/BL6J-βENaC-Tg mice. Representative data of PAS and alcian blue-stained section of WT (**a**) (*n* = 9) and C57/BL6J-βENaC-Tg (**b**) (*n* = 11) mice. Square diameter, 300 μm. Quantitative morphometric analysis of alveolar septae of the lungs is shown in (**c**). (**d**–**i**) Respiratory parameters (resistance, elastance, compaliance, FVC, FEV0.1 and FEV0.1%) of WT (*n* = 15) and C57/BL6J-βENaC-Tg (*n* = 17) mice analyzed by flexiVent. ^**^p < 0.01, ^***^p < 0.001, versus WT; Student’s *t* test. (**j**,**k**) Correlation analysis of parameters. Summary of analysis between pulmonary parameters and biochemical parameters in BALF in WT and C57/BL6J-βENaC-Tg mice (*n* = 36–41) (**j**). ^*^p < 0.05; Pearson’s correlation coefficient test. Correlation scatter plots of MLI and FEV0.1% (**k**).

To further characterize the pulmonary phenotypes of C57/BL6J-βENaC-Tg mice, we next determined the alveolar mean linear intercept (MLI), the most common morphometric method to assess emphysema in animal models. Importantly, C57/BL6J-βENaC-Tg mice had significantly higher MLI length (Fig. 2a–c), indicating the spontaneous emphysematous phenotype in C57/BL6J-βENaC-Tg mice, as was also shown in previous investigations^6^,^7^. We next determined the pulmonary mechanics and function of C57/BL6J-βENaC-Tg mice. Clinically acceptable respiratory parameters, such as resistance (R), elastance (E), compliance (C = 1/E), forced vital capacity (FVC), forced expiratory volume in 0.1 second (FEV0.1) and FEV0.1% (FEV0.1/FVC), were analyzed by invasive lung function measurements using the flexiVent system. Among the mechanistic parameters we tested, airway elastance and compliance were significantly decreased and increased, respectively, in C57/BL6J-βENaC-Tg mice (Fig. 2d–f). Moreover, pulmonary functional markers FVC and FEV0.1 were significantly increased, while FEV0.1/FVC, a marker of airflow obstruction during expiration, was significantly decreased in C57/BL6J-βENaC-Tg mice (Fig. 2g–i), suggesting the impaired pulmonary mechanics and function in our established βENaC-Tg mice. We next determined which pulmonary parameters are strongly associated with mucus overproduction and inflammatory biochemical parameters by correlation analysis. Importantly, among the pulmonary histological and mechanical parameters, MLI and FEV0.1/FVC were well correlated with Fucose, MUC5AC and KC levels in BALF of βENaC-Tg mice (Fig. 2j; Supplementary Fig. 1). Moreover, the values of MLI and FEV0.1/FVC were also well correlated in individuals (Fig. 2k), indicating that MLI and FEV0.1/FVC in βENaC-Tg mice can be considered as the ideal parameters that meet the criteria of obstructive pulmonary diseases.

### DNA microarray analysis in lung tissue of C57/BL6J-βENaC-Tg mice

**Figure 3:**
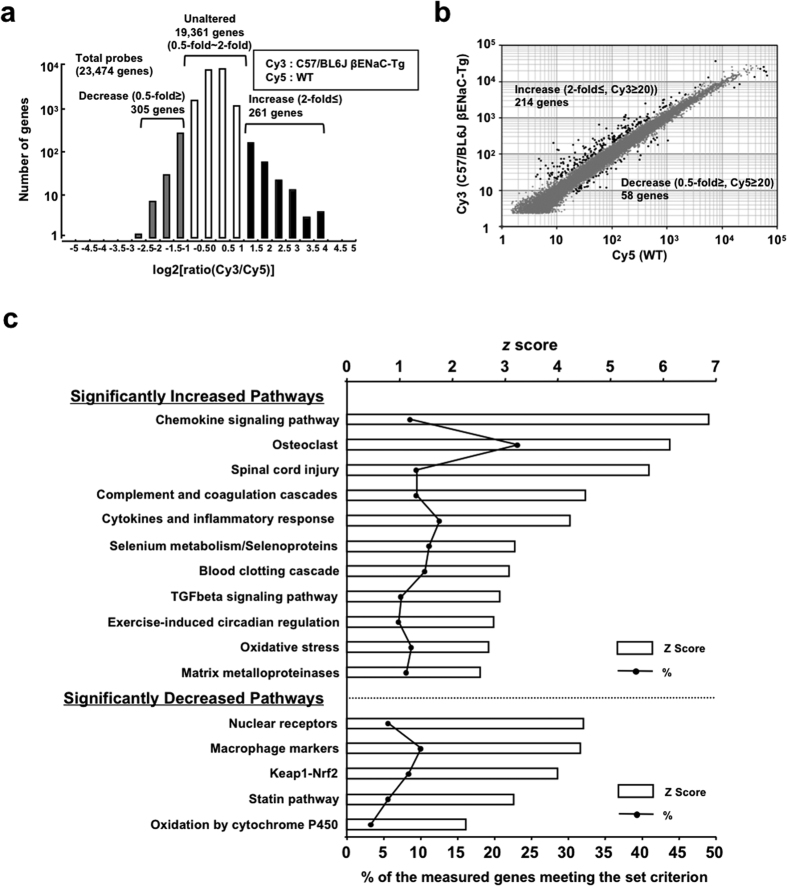
Microarray analysis of lung mRNA of WT and in C57/BL6J-βENaC-Tg mice. (**a**) A histogram representing the distribution of log2[ratio Cy3(C57/BL6J-βENaC-Tg)/Cy5 (WT)]. Number of genes up-regulated (log2[ratio Cy3/Cy5] ≥ 1.0) and down-regulated (log2[ratio Cy3/Cy5] ≤ −1.0) in lung tissue of C57/BL6J-βENaC-Tg mice is indicated. (**b**) Scatter plots based on raw values of significantly up-regulated and down-regulated meaningful genes in C57/BL6J-βENaC-Tg mice are indicated as closed circles (cut-off value was set). (**c**) Statistically significantly increased and decreased pathways in C57/BL6J-βENaC-Tg mice are listed.

To characterize the pulmonary phenotypes of C57/BL6J-βENaC-Tg mice at the molecular level, we isolated total RNA from lung tissues of WT and C57/BL6J-βENaC-Tg mice and performed DNA microarray analysis by the “3D-Gene” mouse oligo chip 24 k (Toray Industries), and ratio (fold induction) of gene expression levels in lung tissue between two genotypes (WT vs. C57/BL6J-βENaC-Tg) was measured. Among the 23,474 genes probed in the chip, expression levels of 19,361 genes were not altered among the mice (ratio between 0.5-fold and 2-fold). On the other hand, 261 genes were up-regulated (2-fold≤) and 305 genes were down-regulated (0.5-fold≥) in lung tissue of C57/BL6J-βENaC-Tg mice (Fig. 3a). We next set cut-off value of Cy3 (C57/BL6J-βENaC-Tg) ≥20 and Cy5 (WT) ≥20 for increased and decreased genes, respectively, which helps to extract realistically altered and meaningful genes. We finally extracted 261 up-regulated genes and 58 down-regulated genes (Fig. 3b; Supplementary Tables 1 and 2). Importantly, expression of some of the up-regulated genes (ex. Il8ra, Saa, Mmp8, Lcn2, Edn1, Ltf, Cp, Ccl2)^15^,^16^,^17^,^18^,^19^,^20^,^21^,^22^,^23^,^24^,^25^,^26^,^27^,^28^,^29^ in C57/BL6J-βENaC-Tg mice were also reported to be increased at similar patterns with both COPD and CF lung tissues (Supplementary Tables 1,2). Moreover, many, but not all, of the genes (11.2% of 214 increased genes, Supplementary Table 1) including Acp5, Ccl6, Clca3, Cd68, Cd84, Ch25h, Chi3l4, Chi3l3, Ctsd, Ctsk, F7, Gpnmb, Itgax, Lilrb4, Lrp12, Ly75, Mcoln3, Mmp12, Ms4a8a, Reg3g, Retnla, Saa3, Slc39a2 and Tbxas1 were also up-regulated; while a few genes (3.4% of 58 decreased genes, Supplementary Table 2) such as Cyp2a4 and Fabp1 were also down-regulated in the lung tissue of βENaC-Tg mice as was the case with previous microarray-based analysis^30^,^31^, implying that our C57/BL6J-βENaC-Tg mice have in part similar molecular characteristics to those of previously established βENaC-Tg mice. To gain insight into the underlying pathophysiology of differentially expressed genes in C57/BL6J-βENaC-Tg mice, pathway analyses were performed. The data on statistically significantly increased and decreased pathways showed the involvement of inflammation-related pathways (Chemokine signaling pathway, Cytokines and inflammatory response, TGFβ signaling pathway), proteases-activated pathways (Osteoclast, Complement and coagulation cascade, Blood clotting cascade, matrix metalloproteinases) and oxidative stress-associated pathways (Oxidative stress, Keap-1-Nrf2, Oxidation by cytochrome P450) (Fig. 3c), which are also known to be associated in CF and COPD pathogenesis^1^,^2^,^3^,^4^. Taken together, these pulmonary biochemical, histological and functional analysis, and comprehensive microarray analysis support the idea that C57/BL6J-βENaC-Tg mice are a good animal model that mimics the molecular pathogenesis of COPD and/or CF.

### Protease inhibitor ONO-3403 suppresses emphysematous phenotype and pulmonary dysfunction in C57/BL6J-βENaC-Tg mice

**Figure 4:**
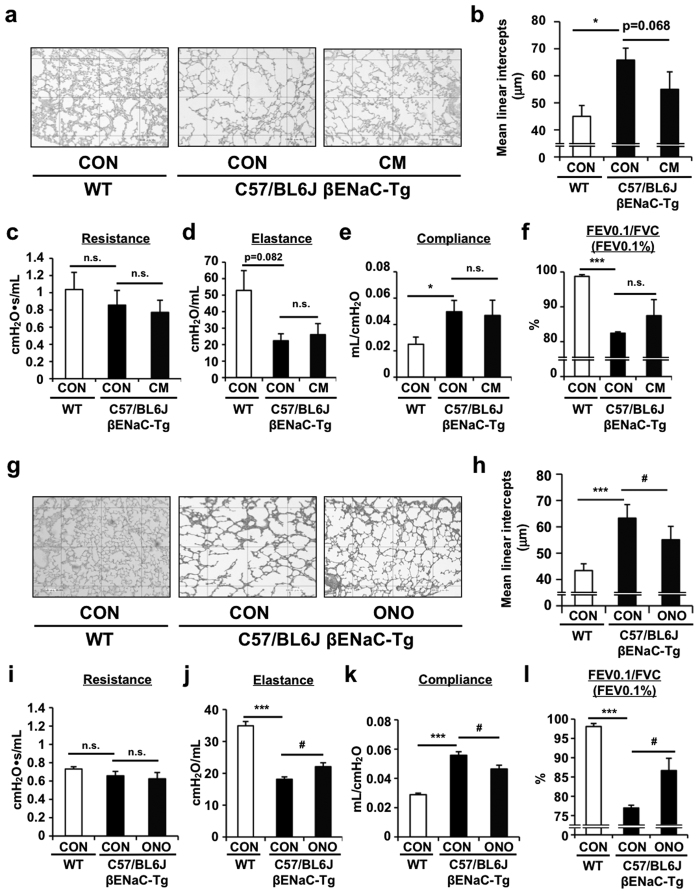
Effect of protease inhibitors on the pulmonary emphysema and dysfunction in C57/BL6J-βENaC-Tg mice. Pulmonary phenotypes were evaluated in oral camostat methylate (CM)- (**a**–**f**) or ONO-3403-treated (**g**–**l**) C57/BL6J-βENaC-Tg mice. Emphysematous phenotypes (**a**,**b**,**g**,**h**) and pulmonary dysfunction (**c**–**f**,**i**–**l**) of the mice were evaluated. Age-matched C57/BL6J mice (WT) were used as healthy controls. *n* = 4–7 and *n* = 7–8 for CM and ONO-3403 treatments, respectively. ^*^p < 0.05, ^***^p < 0.001, versus WT mice; ^#^*p* < 0.05, versus vehicle-treated C57/BL6J-βENaC-Tg mice; Student’s *t* test.

Since DNA microarray analysis revealed that protease-antiprotease imbalance is present in the lung tissue of βENaC-Tg mice and ENaC is basically activated by channel-activating serine proteases (CAPs)^2^, we determined if the treatment with selective serine protease inhibitors is beneficial to C57/BL6J-βENaC-Tg mice. Two orally active inhibitors including camostat mesilate (CM) and its derivative ONO-3403, a synthetic serine protease inhibitor that has a higher protease-inhibitory activity compared with CM, were administered to C57/BL6J-βENaC-Tg mice and the effect on emphysematous phenotype and pulmonary dysfunction was determined^32^,^33^. CM treatment had no beneficial effects on emphysema (Fig. 4a,b), pulmonary mechanics such as resistance, elastance and compliance (Fig. 4c–e), and pulmonary function (Fig. 4f; Supplementary Fig. 2). ONO-3403 significantly improved emphysematous phenotype and pulmonary dysfunction (Fig. 4g–l; Supplementary Fig. 2) in C57/BL6J-βENaC-Tg mice, suggesting that massive intervention of proteases-activation pathways is effective in improving emphysematous phenotype and pulmonary dysfunction in C57/BL6J-βENaC-Tg mice.

### Cluster analysis of ONO-3403-treated C57/BL6J-βENaC-Tg mice

To ascertain which pathways are affected by ONO-3403 treatment in C57/BL6J-βENaC-Tg mice, microarray-based cluster analysis was performed. Based on the patterns of gene alteration in lung tissue of ONO-3403-treated and -untreated C57/BL6J-βENaC-Tg mice compared to WT mice, a total of five cluster sets were determined (Supplementary Fig. 3a,b). Cluter I and IV were the gene sets that are significantly up-regulated or down-regulated in C57/BL6J-βENaC-Tg mice but are not affected by ONO-3403 treatment. Cluster II and III were significantly up-regulated and suppressed by ONO-3403 treatment, while Cluter V was significantly down-regulated and recovered to normal level by ONO-3403 treatment (Supplementary Fig. 3a,b). These cluster analyses demonstrated that ONO-3403 improves many of the dysregulated gene expression (Supplementary Fig. 3a,b). Pathway analysis further revealed that the COPD- and CF-associated pathways, such as inflammation-related, proteases-activated and oxidative stress-associated pathways, dominantly consist of Cluster II, III and V (Supplementary Fig. 3b,c), confirming the critical role of protease-antiprotease imbalance in the pathogenesis of C57/BL6J-βENaC-Tg mice.

### Intratracheal administration of N-acetylcystein suppresses emphysematous phenotype and pulmonary dysfunction in C57/BL6J-βENaC-Tg mice

**Figure 5:**
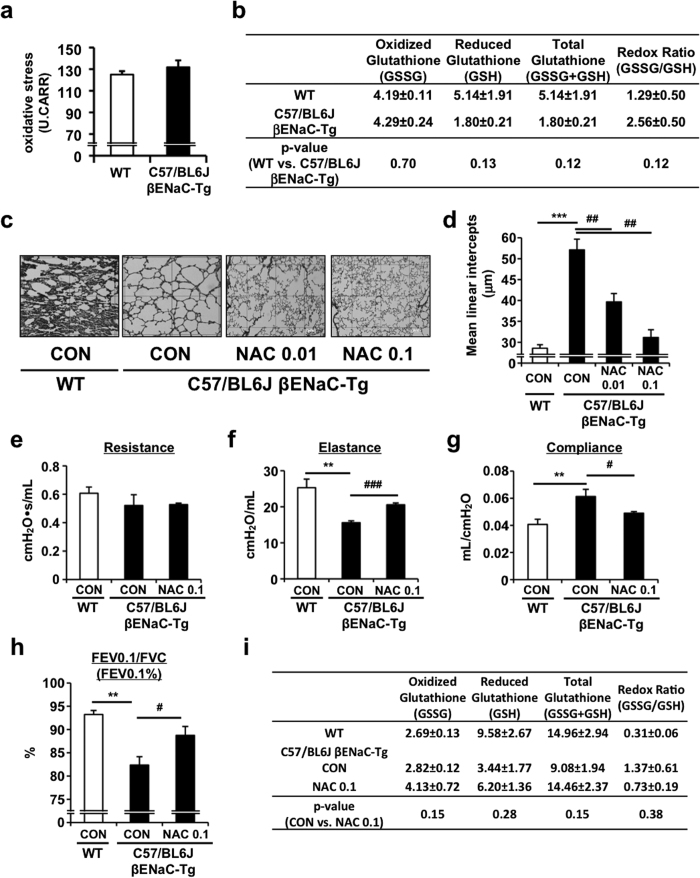
Effect of N-acetylcysteine on the pulmonary emphysema and dysfunction in C57/BL6J-βENaC-Tg mice. (**a**) Serum levels of oxidative stress in WT and C57/BL6J-βENaC-Tg mice. *n* = 5 mice/group. (**b**) Oxidative stress in BALF of WT and C57/BL6J-βENaC-Tg mice were evaluated by the levels of glutathiones (GSSG, GSH and total glutathione). *n* = 4 mice/group. GSSG/GSH was shown as a quantitative marker of oxidative stress. P-value versus WT mice were assessed by Student’s *t* test. (**c**,**d**) Pulmonary phenotypes were evaluated in intratracheal N-acetylcysteine (NAC)-treated C57/BL6J-βENaC-Tg mice. Emphysematous phenotypes of the mice with NAC treatments (0.01 and 0.1 mg kg^−1^) were evaluated. (**e**–**h**) Pulmonary dysfunction of the mice with NAC treatments (0.1 mg kg^−1^) was evaluated. Age-matched C57/BL6J mice (WT) were used as healthy controls. *n* = 4-5 mice/group. ^**^p < 0.01, ^***^p < 0.001, versus WT mice; Student’s *t* test. ^#^*p* < 0.05, ^##^*p* < 0.01, ^###^*p* < 0.001 versus vehicle-treated C57/BL6J-βENaC-Tg mice; Dunnett’s test. (**i**) The levels of glutathiones (GSSG, GSH and total glutathione) in BALF of WT, C57/BL6J-βENaC-Tg mice treated with or without NAC (0.1 mg kg^−1^). *n* = 3 mice/group. P-value versus NAC-nontreated C57/BL6J-βENaC-Tg mice were assessed by Student’s *t* test.

Because our microarray analysis also implied an imbalance between oxidants and antioxidants in C57/BL6J-βENaC-Tg mice, we determined the status of whole body or local oxidative stresses in C57/BL6J-βENaC-Tg mice. Serum oxidative stress was not altered in C57/BL6J-βENaC-Tg mice (Fig. 5a), but the levels of reduced and total glutathiones (GSH and GSSG+GSH), indicators of anti-oxidative capacity, were decreased in accordance with an increase in the redox ratio (GSSG/GSH) in BALF, confirming the oxidative stress in the pulmonary tissue of C57/BL6J-βENaC-Tg mice (Fig. 5b). To further characterize the importance of anti-oxidative glutathione, rescue experiments were performed by intratracheal instillation of N-acetylcysteine (NAC), a precursor of glutathione^34^. Notably, NAC treatment significantly suppressed emphysematous phenotype (Fig. 5c,d). Moreover, NAC improved pulmonary mechanics (Fig. 5e–g) and function (Fig. 5h; Supplementary Fig. 4). Consistently, the amount of anti-oxidative glutathiones as well as the redox ratio (GSSG/GSH) in BALF were recovered to the normal levels by NAC treatment (Fig. 5i), supporting the idea that pulmonary oxidative stress, at least in part, contributes to the emphysematous phenotype and pulmonary dysfunction in C57/BL6J-βENaC-Tg mice.

### Deficiency of endogenous vitamin C synthesizing enzyme dampens COPD/CF-like symptoms of C57/BL6J-βENaC-Tg mice

**Figure 6:**
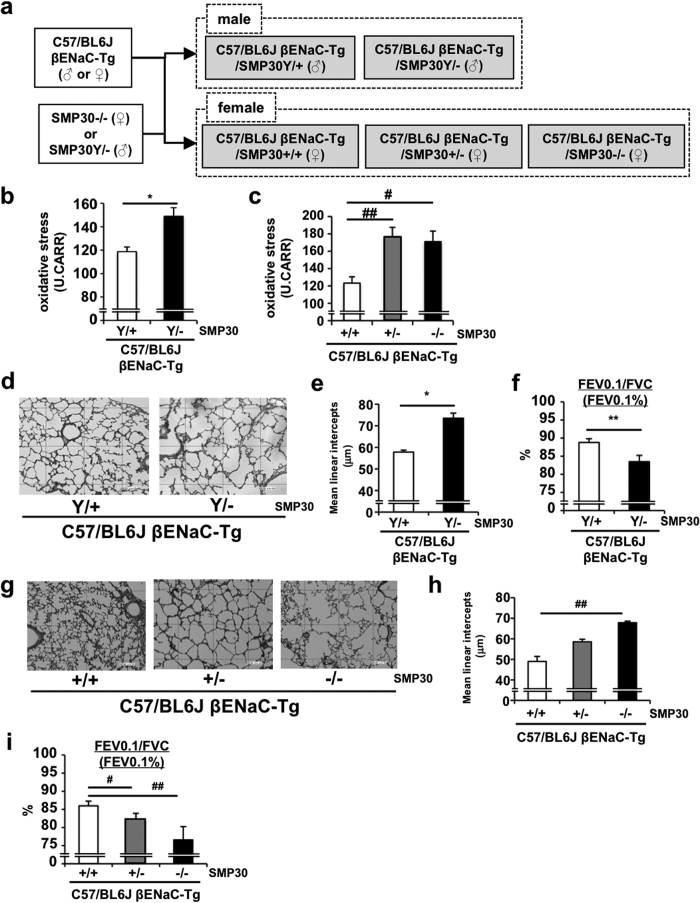
SMP30 deficiency exacerbates pulmonary emphysema and dysfunction in C57/BL6J-βENaC-Tg mice. (**a**) Scheme of the generation of C57/BL6J-βENaC-Tg-SMP30 KO mice. (**b**,**c**) Serum levels of oxidative stress in C57/BL6J-βENaC-Tg and C57/BL6J-βENaC-Tg-SMP KO mice ((**b**), male; (**c**), female). *n* = 3–7 mice/group. ^*^p < 0.05, versus C57/BL6J-Tg (Y/+) mice; Student’s *t* test. ^#^*p* < 0.05, ^#^*p* < 0.01, versus C57/BL6J-βENaC-Tg (+/+) mice; Dunnett’s test. (**d**–**i**) Pulmonary phenotypes were evaluated in male (**d**–**f**) and female (**g**–**i**) C57/BL6J-βENaC-Tg and C57/BL6J-βENaC-Tg-SMP KO mice. Emphysematous phenotypes (**d**,**e**,**g**,**h**) and pulmonary dysfunction (**f**,**i**) of the mice were evaluated. *n* = 6 mice/group. ^*^p < 0.05, ^**^p < 0.01, versus C57/BL6J-βENaC-Tg (Y/+) mice; Student’s *t* test. ^#^*p* < 0.05, ^##^*p* < 0.01, versus C57/BL6J-βENaC-Tg (Y/+) mice; Dunnett’s test.

Based on the fact that plasma concentration of vitamin C (VC), one of the strongest anti-oxidants, is significantly decreased in COPD patients^35^ and is inversely correlated with pulmonary inflammatory phenotype in CF patients^36^, we sought to investigate whether VC could modulate pulmonary phenotypes of C57/BL6J-βENaC-Tg mice. We first crossed C57/BL6J-βENaC-Tg mice with senescence marker protein-30 (SMP30) knockout (KO) mice, which have been shown unable to synthesize VC endogenously (Fig. 6a)^37^, and utilized C57/BL6J-βENaC-Tg-SMP30 KO mice deprived of VC for 8 weeks. Under VC depletion condition, serum oxidative stress was increased in both male and female C57/BL6J-βENaC-Tg-SMP30 KO mice (Fig. 6b,c). Notably, emphysematous phenotype and pulmonary dysfunction were further exacerbated with SMP30 deficiency in both male and female mice (Fig. 6d–i; Supplementary Fig. 5), probably due to a decreased VC concentration and an increased oxidative stress in serum. These results suggest that anti-oxidant VC has a protective role against COPD/CF-like pulmonary phenotypes in C57/BL6J-βENaC-Tg mice, which is consistent with the clinical and pathophysiological relevance of VC in COPD and CF patients.

### Effect of serine protease inhibition and VC depletion on the expression levels of mucus-related and inflammatory genes in C57/BL6J-βENaC-Tg mice

**Figure 7:**
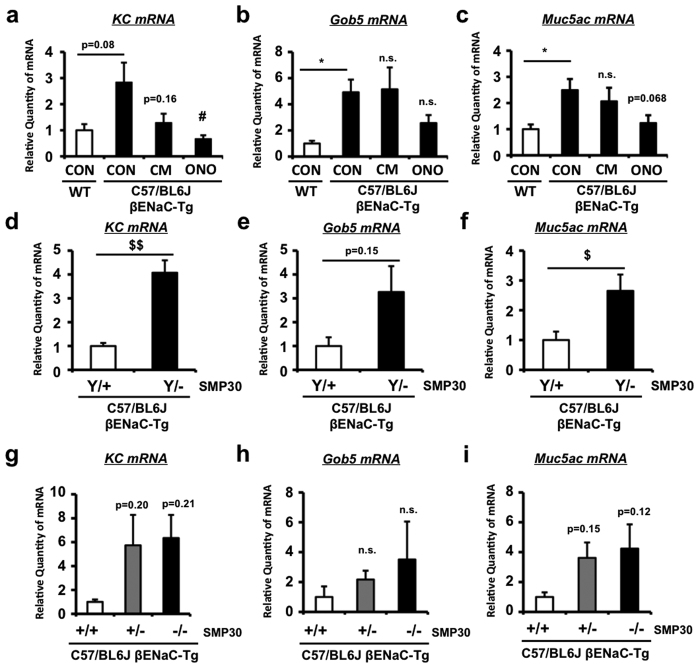
Effect of serine protease inhibition and VC depletion on the expression levels of mucus-related and inflammatory genes in C57/BL6J-βENaC-Tg mice. Pulmonary mRNA expression of *KC* (**a**,**d**,**g**), *Gob5* (**b**,**e**,**h**) and *Muc5ac* (**c**,**f**,**i**) genes in WT and C57/BL6J-βENaC-Tg mice treated with vehicle (CON), CM or ONO-3403 (**a**–**c**) (*n* = 6–7 mice/group), and C57/BL6J-βENaC-Tg and C57/BL6J-βENaC-Tg-SMP KO mice ((**d**–**f**), male; (**g**–**i**), female) (*n* = 4–6 mice/group) quantified by Q-RT-PCR. ^*^p < 0.05, versus WT; Student’s *t* test. ^#^*p* < 0.05 versus vehicle-treated C57/BL6J-βENaC-Tg mice; Dunnett’s test. ^$^*p* < 0.05, ^$$^*p* < 0.01, versus C57/BL6J-βENaC-Tg (Y/+) or (+/+) mice; Dunnett’s test.

Finally, to confirm whether serine protease inhibition and VC depletion also affect mucus overproduction and inflammatory biochemical parameters, we evaluated the gene expression levels of mucus-associated and neutrophil-dominated inflammatory genes in lung tissues of WT and C57/BL6J-βENaC-Tg mice. Serine protease inhibition by ONO-3403, but not CM, significantly decreased KC expression and tended to decrease Muc5ac expression (Fig. 7a–c), suggesting that ONO-3403-dependent improvement of pulmonary dysfunction and emphysema is probably due to the lowering effect on KC and Muc5ac gene expression. Moreover, VC deficiency by SMP30 depletion in C57/BL6J-βENaC-Tg mice significantly increased KC and Muc5ac expression in male (Fig. 7d–f), while tended to, but not significantly, increase KC and Muc5ac expression in female in a gene dose-dependent manner (Fig. 7g–i). These data may support the idea that serine protease inhibition and VC depletion affect pulmonary function and emphysematous phenotype partly through their effects on mucus overproductive and inflammatory biochemical characteristics of C57/BL6J-βENaC-Tg mice.

## Discussion

In the present study, we have established and characterized low-mortality airway-specific βENaC-Tg mice with C57/BL6J background as a possible model of COPD/CF lung diseases. Reduced mortality is consistent with the previous findings on improved mortality of C57BL/6 backcrossing in βENaC-Tg mice^13^,^14^. Importantly, despite a reduction in mortality, the mice maintained mucus obstructive, airway inflammatory and emphysematous phenotypes. Because, until now, only limited laboratories showed the usefulness of βENaC-Tg mice, the similar findings from independent groups strongly imply the stable application of βENaC-Tg mice in the fields of COPD and CF researches. Because correlation analysis revealed that MLI and FEV0.1% of C57/BL6J-βENaC-Tg mice are reliable parameters to represent other pulmonary phenotypes (Fig. 2j,k; Supplementary Fig. 1), and these parameters were altered by pharmacological and genetic approaches targeting proteases- and oxidative stress-pathways that are typically activated in COPD/CF lung^1^,^2^,^3^,^4^, C57/BL6J-βENaC-Tg mice could be a clinically promising animal model to discover the novel targets of COPD/CF lung diseases.

However, whether the C57/BL6J-βENaC-Tg mice are mechanistically identical to COPD and CF lung diseases needs to be clarified. As indicated in the number of clinical studies, pathogenesis of COPD is strongly linked to the effects of cigarette smoke (CS) on the lungs^1^,^2^,^38^. In this connection, CS and CS-related proteases (elastase, papain) exposure models have been widely used in the field of COPD basic research^39^, but these models lack the inducing ability of mucus obstructive phenotype, a critical symptom that affects the frequency of COPD exacerbation and patients’ quality of life^40^. Notably, Seys *et al*. showed the direct modulation of airway inflammation, mucin expression and emphysematous phenotypes by CS exposure in βENaC-Tg mice^41^. On the other hand, recent report revealed that CS exposure results in a hyposecretory/hyperabsorptive ion transport phenotype by down-regulating CFTR, a crucial cAMP-dependent Cl^−^ channel that is mutated in CF^42^,^43^,^44^, and by directly or indirectly enhancing the ENaC activity in airway epithelial cells^45^,^46^. The fact of CS-dependent ENaC activation (CS-ENaC axis) in COPD patients insures that βENaC-overexpressing mice is a meaningful causative model of COPD^46^. As for CF, the concept of enhanced ENaC activity due to the reduction of CFTR function is widely accepted, which is the reason why βENaC-Tg mice was originally produced^5^,^9^,^10^. Overall, these lines of evidence support the idea that C57/BL6J-βENaC-Tg mice, with or even without CS-exposure, are useful models of COPD/CF that have low-mortality and spontaneously exhibit obvious mucus obstructive phenotype.

Our data on the beneficial effects of serine protease inhibitor may provide clinical relevance on targeting protease pathways for the treatment of COPD/CF. Although protease-antiprotease imbalance is widely known as one of the major causes that induce emphysematous phenotype in COPD/CF patients^2^,^12^, protease inhibitors have yet to be clinically available. Until now, inhibitor of neutrophil elastase, or Sivelestat (ONO-5046), has been approved in Japan for clinical use in the treatment of acute lung injury (ALI)/acute respiratory distress syndrome (ARDS)^47^. However, development of Sivelestat was unsuccessful and discontinued in USA^48^, suggesting that specific inhibition of neutrophil elastase may not be beneficial to COPD and CF patients, which is also supported by the finding that depletion of neutrophil elastase in βENaC-Tg mice does not improve mucus obstructive phenotype^49^. Moreover, we and others reported that expression of another type of protease, or MMP-12, is increased in βENaC-Tg mice (Supplementary Table 1)^30^. However, MMP-12 depletion was only beneficial to emphysematous phenotype, but not mucus obstructive phenotype^30^. On the other hand, in the pathogenesis of COPD and CF, proteases activation is not only related to inflammatory responses but also to hyposecretory/hyperabsorptive ion transport phenotype, or mucus obstructive phenotype in airway epithelial cells^2^. Because camostat methylate (CM) and CM-derivative ONO-3403 has been shown to selectively inhibit trypsin-like serine proteases, such as channel-activating proteases (CAPs) that is known to cleave ENaC channel subunits and increase ENaC activity^32^,^33^, ENaC inhibition by targeting CAPs, but not neutrophil elastase, may be a better approach based on the beneficial effect of ONO-3403 in this study (Fig. 4g–l; Supplementary Fig. 2). On the other hand, there was the notion that a CAP inhibitor is not expected to modulate ENaC activity in βENaC-Tg mice due to protease-independent constitutive activation of ENaC channel in this model^50^. Consistently, supplemental analysis revealed that heterozygous deletion of CAP-1/prostasin/Prss8, one of the major CAPs that positively regulates ENaC, did not improve COPD/CF-like phenotypes such as emphysema and lung dysfunction (Supplementary Fig. 6). Although “ENaC-targeting concept” is about to be confirmed by the recent basic report^51^ and the clinical study for the treatment of CF on a long-acting inhaled ENaC inhibitor, P1037 (VX371) (Phase 2 CLEAN-CF), beneficial effects of ONO-3403 on C57/BL6J-βENaC-Tg mice may be exerted by ENaC-independent unknown mechanisms.

Another important characteristic of C57/BL6J-βENaC-Tg mice is the oxidative stress-antioxidant imbalance. It is highly likely that higher endogenous oxidative stress of C57/BL6J-βENaC-Tg mice is detrimental, so that glutathione precursor anti-oxidant NAC had a beneficial effect on the pulmonary phenotypes of C57/BL6J-βENaC-Tg mice. This efficacy of NAC in C57/BL6J-βENaC-Tg mice is identical to what was observed in several reports that show the effect of NAC in the improvement of small airways function and the decrease of exacerbation frequency in COPD patients^52^ as well as in the attenuation of lung dysfunction in CF patients^53^. These similarities of drug responsiveness further emphasize that C57/BL6J-βENaC-Tg mice is useful for COPD/CF researches. Moreover, our study also revealed the importance of endogenous anti-oxidant as depletion of endogenous anti-oxidant VC in C57/BL6J-βENaC-Tg mice (SMP30 knockout with VC depletion in the food) further exacerbates pulmonary phenotype. This result also reflects our previous study on SMP30 knockout in CS-induced emphysema model^54^. Because (i) plasma VC level is decreased and VC supplementation is generally recommended in CF and COPD patients and ii) VC is not endogenously produced in human but produced in mice^37^, our study supports the idea that C57/BL6J-βENaC-Tg-SMP30 KO mice may be one of the ideal CF and COPD models that mimic the characteristics of human patients with lower VC level.

Finally, a key issue that has yet to be discussed is the efficacy of therapeutic interventions by our and others’ genetic and pharmacological studies on βENaC-Tg mice. Zhou *et al*. originally described the importance of early and continuous but not late and temporary inhibitions of ENaC activity by amiloride in the suppressive effect on all phenotypes of βENaC-Tg mice, indicating that transitory ENaC activation is critical to initiate disease development, especially in severe type of βENaC-Tg mice model, and targeting ENaC channel alone in adulthood may not be sufficient to cure all symptoms^55^. Livraghi *et al*. showed the indispensable role of IL-4R- and TNF-α-dependent increases in the physiological regulation of mucous secretory cell (MuSC) and eosinophils, but the pathways were dispensable for the regulation of neutrophilia, mucus obstruction and airspace enlargement^56^. Consistently, inhibition of infection-associated inflammation by the depletion of MyD88, a critical adaptor molecule that mediates a number of toll-like receptors signaling, did not seem to be sufficient to control mucus obstructive phyenotypes^57^. These findings imply that ENaC over-activation *per se* in airway causes inflammation, but anti-inflammatory targeting has little impact on COPD-like pathogenesis. On the other hand, the fact that targeting surface dehydration by inhaled hypertonic saline (HS), an inducer of osmotic water flux, improves not only dehydration of airway surfaces but also pulmonary mortality and airway mucus obstruction despite a minor effect on inflammation, further supports the idea of mucus obstruction and inflammation as independent pathways^58^. Interestingly, a recent report demonstrated that inhibition of PI3Kγ decreases both neutrophilic airway inflammation and structural lung damage in βENaC-Tg mice^59^. Moreover, our study has also revealed the beneficial effects of serine protease inhibitors and anti-oxidant drugs on the pulmonary dysfunction and emphysematous phenotype, and of the potential effect on neutrophilic-inflammatory phenotype (KC induction) in C57/BL6J-βENaC-Tg mice. Possible involvement of PI3Kγ-dependent signals on the therapeutic effects of anti-protease and anti-oxidative stress needs to be further studied.

In summary, we produced C57/BL6J-βENaC-Tg mice as a useful murine model of COPD/CF. Numerous assessments including pulmonary histological, biochemical and functional assays in combination with pharmacological and genetic approaches revealed that protease-antiprotease imbalance and oxidative stress are major pathophysiological hallmarks of C57/BL6J-βENaC-Tg mice, which are also known as typical characteristics in human COPD/CF. Since many researchers are starting to pay attention to βENaC-Tg mice as a model of COPD/CF^5^,^12^,^13^,^46^,^49^,^60^, our study provides fundamental information on pulmonary phenotypes of βENaC-overexpressing mice with an emphasis on the usefulness of C57/BL6J-βENaC-Tg mice. Further, the study also provides a reappraisal of the potential effects of serine protease inhibitors and anti-oxidant drugs on obstructive lung diseases such as COPD and CF. Although our analysis could not determine how protease pathway and oxidative stress pathway are mutually connected, the fact that ONO-3403 suppresses both proteases-activated and oxidative stress-associated pathways (Supplementary Fig. 3) supports the idea of feed-forward regulation between proteases-activated and oxidative stress-associated pathways in the pathogenesis of C57/BL6J-βENaC-Tg mice.

## Methods

### βENaC-Tg mice and treatments

To produce low-mortality βENaC-Tg mice, we obtained congenic line of original βENaC-Tg mice (B6.Cg-Tg(Scgb1a1-Scnn1b)6608Bouc/J)^5^ from the Jackson Laboratory (Bar Harbor, Maine) and backcrossed to C57/BL6J for at least three generations. The produced line was named as C57/BL6J-βENaC-Tg mice. The mice were genotyped by manufacturer’s recommendation with the following primer sets; FW 5′-CTTCCAAGAGTTCAACTACCG-3′ and RV 5′-TCTACCAGCTCAGCCACAGTG-3′ (245 bp). In some experiments, reproductive techniques established by Center for Animal Resources and Development (CARD) were applied to efficiently produce an optimal number of mice with stably exhibiting pulmonary phenotypes^61^. The mice were housed in a vivarium in accordance with the guidelines of the animal facility center of Kumamoto University and were fed with normal chow *ad libitum*. For the healthy control group, age-matched wild-type (WT) C57/BL6J mice were used. For the pharmacological treatments of C57/BL6J-βENaC-Tg mice, adult C57/BL6J-βENaC-Tg mice (14–16 weeks old, twice per day) were used. Camostat methylate (CM) and ONO-3403 were obtained from Ono pharmaceutical company Ltd. (Osaka, Japan) and the mice were treated *p.o.* with methylcellulose (vehicle) or 100 mg kg^−1^ CM or 20 mg kg^−1^ ONO-3403 for 2 weeks (ONO-3403) or 3 weeks (CM). N-acetylcystein (NAC) were purchased from Sigma (St. Louis, MO) and the mice were treated with 0.01–0.1 mg kg^−1^ NAC dissolved in PBS for 2 weeks (*i.t.*). All experiments were performed according to the protocols approved by the Animal Welfare Committee of Kumamoto University.

### Producing VC-deficient mice

SMP30-KO mice were established, genotyped and maintained as previously described^37^. C57/BL6J-βENaC-Tg-SMP30 KO mice were produced by crossing C57/BL6J-βENaC-Tg mice and SMP30-KO mice. Because SMP30 is X-linked gene, male mice were genotyped as SMP30 WT homozygotes (Y/+) and KO hemizygotes (Y/−), while female mice were genotyped as SMP30 WT homozygotes (+/+), heterozygotes (+/−) and KO homozygotes (−/−). For the maintenance of SMP30 lines, the mice were given with physiologically sufficient VC (1.5 g l^−1^ VC) in drinking water. For the induction of VC depletion, the mice (8-weeks old) were fed with VC-deficient diet (CL-2, CLEA Japan, Tokyo, Japan) and non-VC-containing drinking water for 8 weeks.

### Producing Prss8+/− mice

Prss8flox/flox mice were originally established as previously described^62^. Prss8+/− mice were obtained by crossing Prss8flox/flox mice and ZP3-cre mice (Bar Harbor, Maine, Jackson laboratory). C57/BL6J-βENaC-Tg-Prss8+/− mice were produced by crossing C57/BL6J-βENaC-Tg mice and Prss8+/− mice.

### RNA isolation, cDNA synthesis and real-time PCR

Quantitative real-time RT-PCR for mouse Scnn1b (βENaC), Gob5, 18 s ribosomal RNA (18srRNA) was carried out based on the previously established methods^63^ using the following primer sets; mouse Scnn1b (FW 5′-ATGTGGTTCCTGCTTACGCTG-3′ and RV 5′-GTCCTGGTGGTGTTGCTGTG-3′), mouse Gob5 (FW 5′-CTGTCTTCCTCTTGATCCTCCA-3′ and RV 5′-CGTGGTCTATGGCGATGACG-3′), mouse KC (FW 5′-TGTCAGTGCCTGCAGACCAT-3′ and RV 5′-CCTCGCGACCATTCTTGAGT-3′), mouse Muc5ac (FW 5′-GTGGTTTGACACTGACTTCCC-3′ and RV 5′-CTCCTCTCGGTGACAGAGTCT-3′) and mouse 18srRNA (FW 5′-GTAACCCGTTGAACCCCATT-3′ and RV 5′-CCATCCAATCGGTAGTAGCG-3′). Briefly, total RNA from the mouse lung tissue was isolated using RNAisoPlus (TaKaRa, Japan) and synthesis of cDNA was performed using PrimeScript RT regent kit (TaKaRa, Japan). Real-time quantitative RT-PCR analysis was performed using SYBR Premix Ex Taq (TaKaRa, Japan) in iQ5 real-time PCR or CFX Connect systems (Bio-Rad, Richmond, CA). The relative quantity of target genes’ mRNA was normalized using mouse 18srRNA as the internal control and expressed as the relative quantity of target genes’ mRNA (fold induction).

### Pulmonary mechanics and function by flexivent

Measurement of pulmonary mechanics (resistance, compliance and elastance) was performed with a computer-controlled small-animal ventilator flexiVent (SCIREQ, Montreal, Canada) as previously described^64^. Briefly, mice were anesthetized with somnopentyl (Kyoritsu, Tokyo, Japan) (15 ml kg^−1^), a tracheotomy was performed, and an 18-gauge needle was inserted into the trachea. Mice were mechanically ventilated at a rate of 150 breaths per min, using a tidal volume of 10 ml kg^−1^ and a positive end-expiratory pressure of 3 cmH_2_O. Pulmonary mechanics were measured by the forced oscillation technique. Resistance represents the level of constriction, while compliance indicates the ease with which the lungs can be extended. Elastance is a reciprocal of compliance that exhibits rigidity of the lungs. Determination of the FEV0.1/FVC ratio was also performed with the flexiVent system connected to a negative pressure reservoir. The ventilated lung was inflated to a pressure of 30 cmH_2_O over 1 s and held at this pressure. After 0.2 s, the pinch valve (connected to ventilator) was closed and after 0.3 s, the shutter valve (connected to negative pressure reservoir) was opened for exposure of the lung to the negative pressure. The negative pressure was held for 1.5 s to ensure complete expiration. Based on the data of forced expiratory volume in 0.1 second (FEV0.1) and forced vital capacity (FVC), pulmonary functional parameter FEV0.1/FVC, or forced expiratory volume % in 0.1 second (FEV0.1%), was determined using the flexiVent software.

### Sample acquisition

After the assessment of pulmonary mechanics and function, mice were sacrificed for the bronchoalveolar lavage fluid (BALF) and histological analysis. The right lobe of lung was lavaged with 0.5 ml of sterile PBS (two times). About 700 μl of BALF was routinely recovered from each animal. Tissue sample of the left lobe of lung was fixed in 10% formalin and then condensed in paraffin using histoprocessor Histos-5 (Milestone, Italy) before being cut into 6 μm thickness. Histological sections were prepared with a rotary microtome Leica RM2125RT (Bensheim, Germany). Samples were first stained with periodic acid-Schiff procedure (PAS) and alcian blue (pH 2.5) to visualize mucus-secreting goblet cells.

### Histological analyses and measurement of MLI

After PAS and alcian blue staining, samples were subjected to hematoxylin and eosin (H&E) staining to visualize in light microscopy for lung morphology. To determine the mean linear intercept (MLI), ten lung sections (3 upper, 4 middle and 3 lower lungs) were selected in an unbiased fashion and 6 lines (300 μm-widths) were drawn randomly on the image of section and the intersection points with the alveolar walls were counted to determine the MLI by dividing the total length of lines drawn across the lung section by the number of intercepts encountered as described in elsewhere^64^.

### BALF analysis

BALF samples were subjected to determine the number of white blood cells and measure the concentration of fucose, Muc5ac, KC and Glutathiones (GSSG, GSH and total Glutathione). The number of leukocytes, neutrophils, lymphocytes and eosinophils were counted by an automatic blood cells counter Sysmex E-2500 (Sysmex, Kobe, Japan). The quantity of fucose in the BALF samples was measured by Winzler’s method^65^. Briefly, 5 ml of 95% ethanol was added to 0.2–0.4 ml of BALF samples, and the centrifuged supernatant was dissolved in 0.5 ml of 0.1 mol l^−1^ NaOH. Cold H_2_SO_4_ solution (2.25 ml) (H_2_SO_4_:H_2_O = 6:1) was added and the tubes were mixed and heated in a boiling water bath for 3 min. After cooling, 0.05 ml of 3% cysteine was added and the tubes were mixed immediately. The tubes were allowed to stand at room temperature for 60–90 min. The absorbance was measured at 396 nm (fucose) and 430 nm (neutral sugar) and the difference in the absorbance values was used for the calculations. L-fucose was used for making a standard curve. To determine the concentration of Muc5ac and KC protein, commercially available human MUC5AC ELISA kit (Cusabio, Wuhan, China) and mouse CXCL1/KC ELISA kit (R&D Systems, Minneapolis, MN) were used, respectively. Concentrations of GSSG, GSH and total Glutathione were measured by using a total glutathione quantification kit (Dojindo, Kumamoto, Japan) by following manufacturer’s instructions.

### Gene expression analysis using DNA microarray

Total RNA was extracted from lung tissues of WT and C57/BL6J-βENaC-Tg mice by RNeasy Mini Kit (QIAGEN, Valencia, CA) and samples were subjected to DNA microarray analysis by the “3D-Gene” mouse oligo chip 24k (Toray Industries Inc, Tokyo, Japan). Ratio (fold induction) of gene expression levels between two genotypes (WT vs. C57/BL6J-βENaC-Tg) was measured based on the signals of Cy5 and Cy3, respectively. Genes with a fold change value ≥2.0 or ≤0.5 were considered to be significantly differentially expressed. For the extraction of the meaningful genes and further analysis, cut-off value of Cy3 (C57/BL6J-βENaC-Tg) ≥20 and Cy5 (WT) ≥20 for increased and decreased genes, respectively, has been used. Pathways affected by βENaC overexpression were determined using the pathway editor, visualization and analysis software PathVisio 3.2.2. The pathways with a Z-score ≥2.0 and a P value < 0.05 were considered to be significantly altered pathways. For the cluster analysis of ONO-3403-responsive genes, RNA samples from ONO-3403-treated C57/BL6J-βENaC-Tg mice were also subjected to “3D-Gene” mouse oligo chip 24k and obtained the fold ratio (fold induction) of gene expression levels compared to WT. By focusing on the genes that are significantly altered in C57/BL6J-βENaC-Tg mice (261 up-regulated and 58 down-regulated genes), cluster analysis were performed by Gene Cluster 3.0 and viewed in a heatmap using Java TreeView 1.1.6r4 software (Stanford University, Palo Alto, CA).

### Evaluation of serum oxidative stress

To measure reactive oxygen metabolites (ROMs), mice serum samples were subjected to d-ROMs test (Diacron srl, Grosseto, Italy), in which the oxidization by hydroperoxyl and alkoxyl radicals, which is originally derived from hydroperoxides by Fenton’s reaction, was quantified photometrically at a wavelength of 505 nm. The d-ROMs test results are expressed as arbitrary units called Carratelli units (CARR U), where 1 CARR U corresponds to 0.08 mg/100 ml H_2_O_2_.

### Statistical analysis

For quantitative analysis, the result represents the mean ± SEM performed in indicated replicates and the data were analyzed by either Student’s *t*-test or one-way ANOVA with Dunnett’s test (JMP software, SAS Institute) as indicated in each figure legend. For correlation analysis, raw data of several parameters in WT and C57/BL6J-βENaC-Tg mice (n = 36–41) were subjected to Pearson’s correlation coefficient test, analyzed by the Statcel 3 program (OMS, Tokorozawa, Japan). The level of significance was set at *p* < 0.05.

## Additional Information

**How to cite this article**: Shuto, T. *et al*. Pharmacological and genetic reappraisals of protease and oxidative stress pathways in a mouse model of obstructive lung diseases. *Sci. Rep.* **6**, 39305; doi: 10.1038/srep39305 (2016).

**Publisher’s note:** Springer Nature remains neutral with regard to jurisdictional claims in published maps and institutional affiliations.

## Supplementary Material

Supplementary Information
