# Supplementary material for: Pharmacological and genetic reappraisals of protease and oxidative stress pathways in a mouse model of obstructive lung diseases
Source: Sci Rep. 2016 Dec 16;6:39305. doi: 10.1038/srep39305 (PMC5159865; doi:10.1038/srep39305)
Supplement: Supplementary Information [file srep39305-s1.pdf]

Supplementary information

Pharmacological and genetic reappraisals of protease and oxidative stress pathways in a mouse model of obstructive lung diseases

Tsuyoshi Shuto[1,*], Shunsuke Kamei[1,2], Hirofumi Nohara[1,2], Haruka Fujikawa[1,2], Yukihiro Tasaki[1], Takuya Sugahara[1], Tomomi Ono[1], Chizuru Matsumoto[1], Yuki Sakaguchi[1], Kasumi Maruta[1], Ryunosuke Nakashima[1], Taisei Kawakami[1], Mary Ann Suico[1], Yoshitaka Kondo[3], Akihito Ishigami[3], Toru Takeo[4], Ken-ichiro Tanaka[5], Hiroshi Watanabe[6], Naomi Nakagata[4], Kohei Uchimura[7], Kenichiro Kitamura[7], Jian-Dong Li[8] and Hirofumi Kai[1,*]

[1]Department of Molecular Medicine, Graduate School of Pharmaceutical Sciences. [2]Program for Leading Graduate Schools "HIGO (Health life science: Interdisciplinary and Glocal Oriented) Program", Kumamoto University, 5-1 Oe-Honmachi, Chuo-ku, Kumamoto 862-0973, Japan. [3]Molecular Regulation of Aging, Tokyo Metropolitan Institute of Gerontology, 35-2 Sakae-cho, Itabashi-ku, Tokyo 173-0015, Japan. [4]Division of Reproductive Engineering, Center for Animal Resources and Development (CARD), Kumamoto University, 2-2-1 Honjo, Chuo-ku, Kumamoto, 860–0811, Japan. [5]Laboratory of Bio-Analytical Chemistry, Research Institute of Pharmaceutical Sciences, Musashino University, Nishitokyo-shi, Tokyo 202-8585, Japan. [6]Department of Biopharmaceutics, Graduate School of Pharmaceutical Sciences, Kumamoto University, 5-1 Oe-honmachi, Chuo-ku, Kumamoto 862-0973, Japan. [7]University of Yamanashi School of Medicine, 1110 Shimokato, Chuo, Yamanashi 409-3898, Japan. [8]Center for Inflammation, Immunity & Infection, Institute for Biomedical Sciences, Georgia State University, 714 Petit Science Center, 100 Piedmont Ave SE, Atlanta, GA30303, USA.

Fig. S1

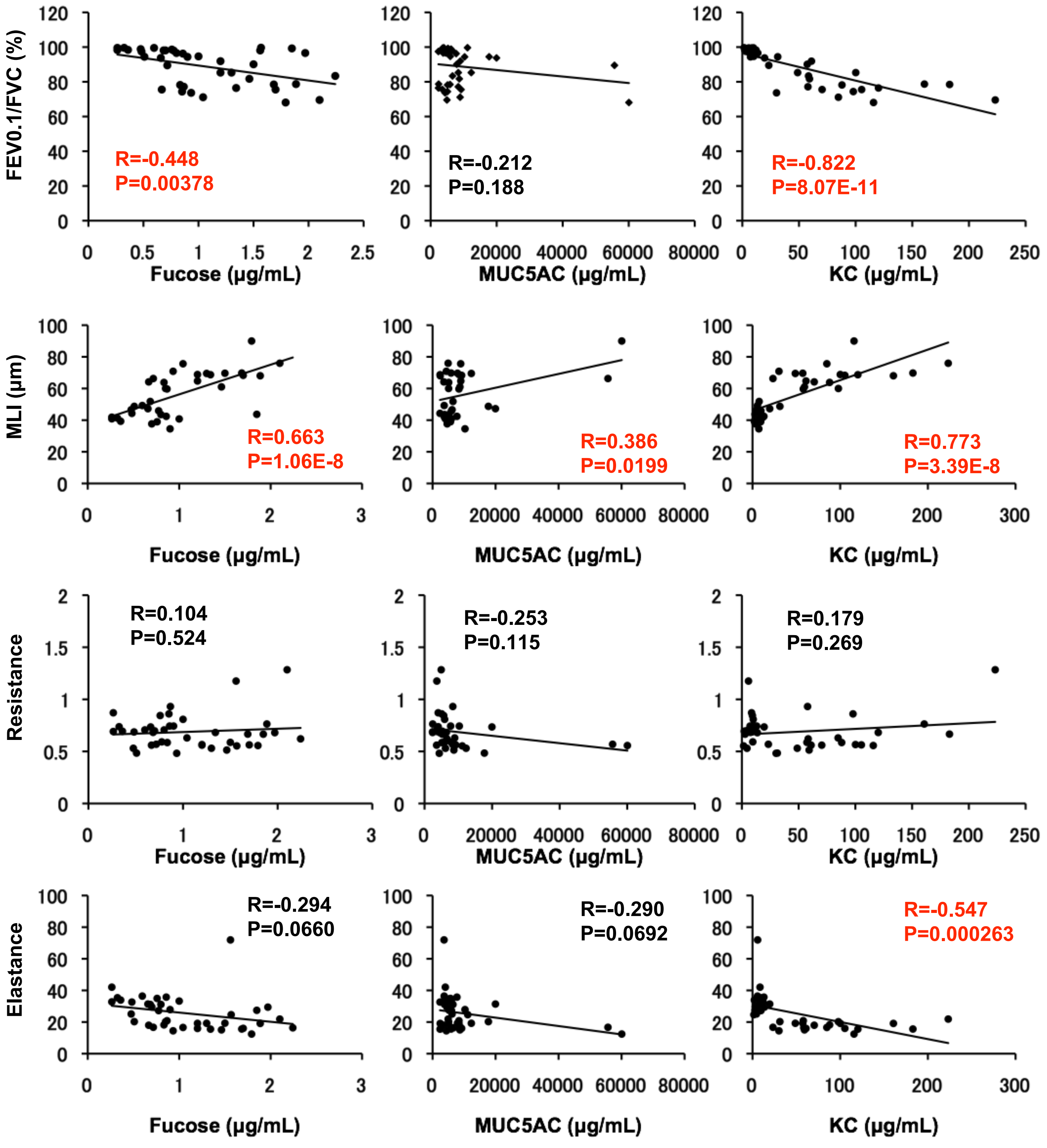

Fig. S1. Correlation analysis of parameters. Analysis between pulmonary parameters and biochemical parameters in BALF in WT and C57/BL6J-βENaC-Tg mice (n=36-41) and correlation scatter plots were shown.

Fig. S2

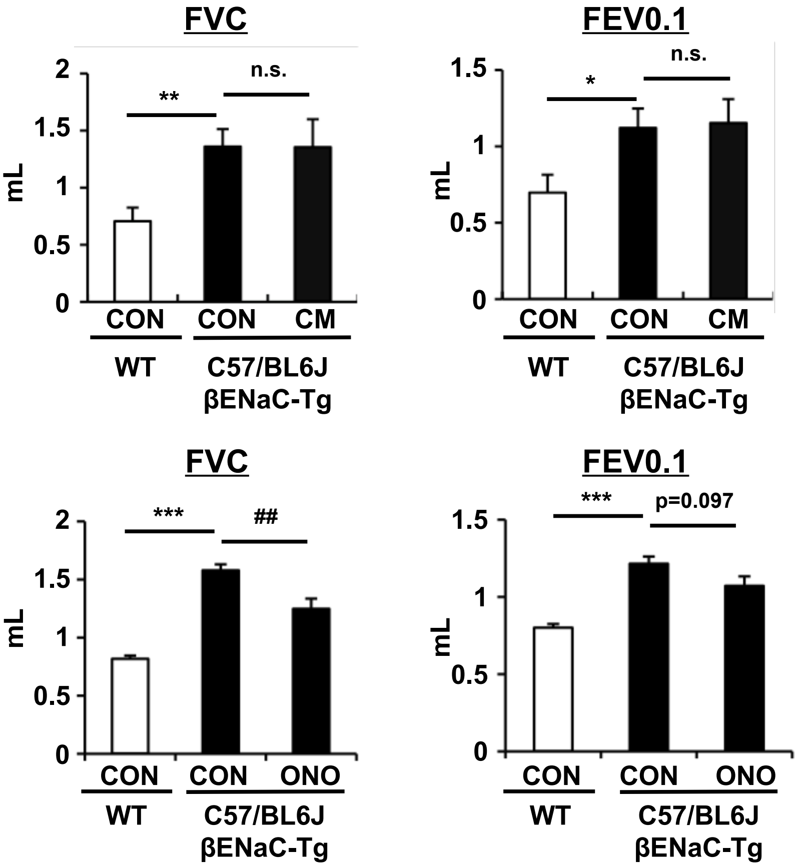

Fig. S2. Effect of protease inhibitors on FVC and FEV0.1 in C57/BL6J-βENaC-Tg mice. Pulmonary phenotypes (FVC and FEV0.1) were evaluated in oral camostat methylate (CM)- or ONO3403-treated C57/BL6J-βENaC-Tg mice. Age-matched C57/BL6J mice (WT) were used as healthy controls. n=4-7 and n=7-8 for CM and ONO3403 treatments, respectively. *$p < 0.05$, **$p < 0.01$, ***$p < 0.001$, versus WT mice; ##$p < 0.01$, versus vehicle-treated C57/BL6J-β ENaC-Tg mice; Student's t test.

Fig. S3

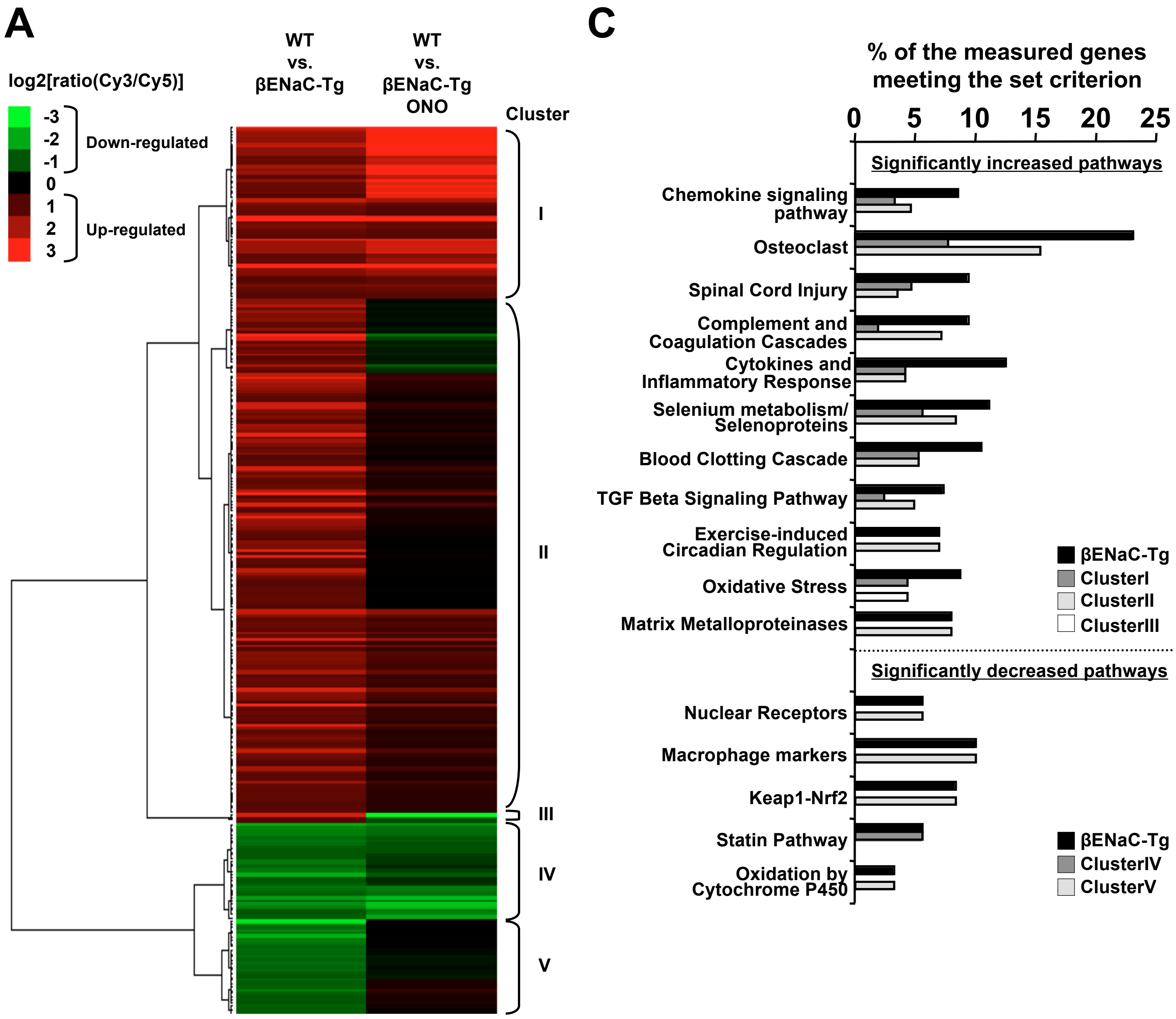

B

	βENaC-Tg	βENaC-Tg ONO	# of genes
Cluster I	+	+ or ++	50
Cluster II	+	± or -	155
Cluster III	+	--	3
Cluster IV	-	- or --	27
Cluster V	-	± or +	28

Fig. S3. Microarray and cluster analyses of ONO3403-treated C57/BL6J-βENaC-Tg mice. (*A*) A heatmap representing the cluster analysis of gene alteration based on the microarray in lung tissue of ONO-3403-treated and -untreated C57BL/6J-βENaC-Tg mice compared to WT mice. Patterns of five cluster sets were indicated in (*B*). +, up-regulated; ++, highly up-regulated; ±, not altered; -, down-regulated; --, highly down-regulated: versus WT mice. (*C*) Comparison of pathway analysis of ONO-3403-treated and -untreated C57BL/6J-βENaC-Tg mice compared to WT mice.

Fig. S4

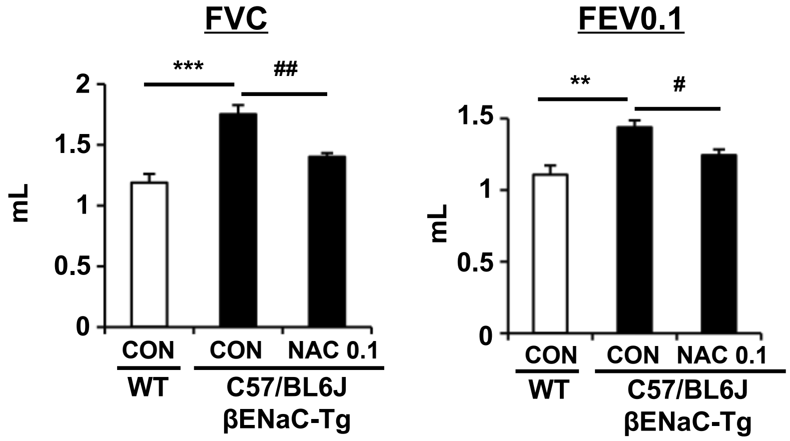

Fig. S4. Effect of NAC on FVC and FEV0.1 in C57/BL6J-βENaC-Tg mice. Pulmonary phenotypes (FVC and FEV0.1) were evaluated intratracheal N-acethylcysteine (NAC)-treated C57/BL6J-βENaC-Tg mice. Age-matched C57/BL6J mice (WT) were used as healthy controls. n=4-5 mice/group. $^{**}p < 0.01$, $^{***}p < 0.001$, versus WT mice; Student's t test. $^{\#}p < 0.05$, $^{\#\#}p < 0.01$, versus vehicle-treated C57/BL6J-βENaC-Tg mice; Dunnett's test.

Fig. S5

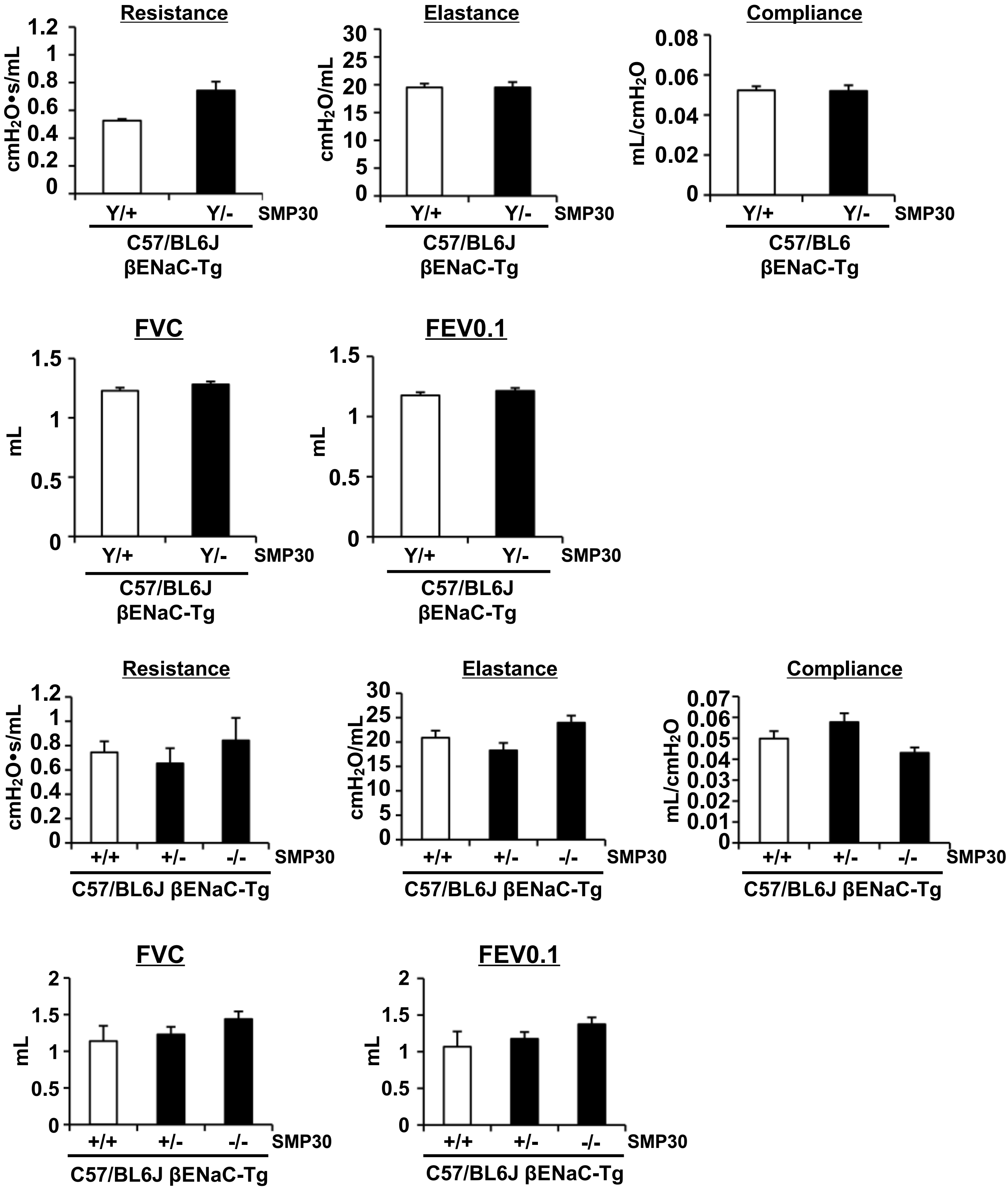

Fig. S5. Effect of SMP30 deficiency on Pulmonary mechanics, FVC and FEV0.1 in C57/BL6J-βENaC-Tg mice. Pulmonary phenotypes (Resistance, Compliance, Elastance, FVC and FEV0.1) were evaluated in male (Y/+ and Y/-) and female (+/+. +/- and -/-) C57/BL6J-βENaC-Tg and C57/BL6J-βENaC-Tg-SMP KO mice. n=6 mice/group.

Fig. S6

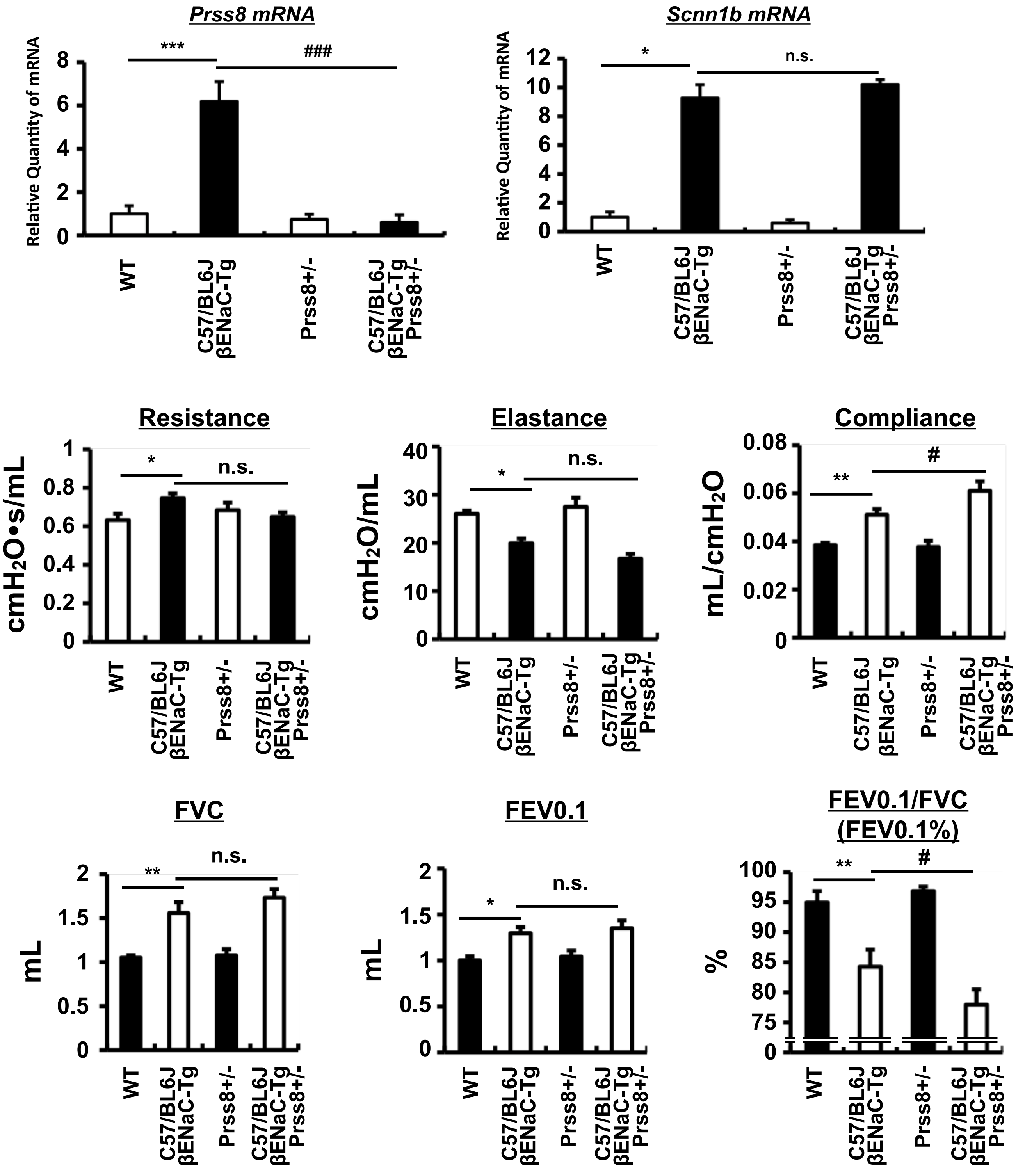

Fig. S6. Effect of heterozygous deletion of Prss8 on genes expression, Pulmonary mechanics, FVC and FEV0.1 in C57/BL6J-βENaC-Tg mice. Gene expression levels of Prss8 and Scnn1b (βENaC) and pulmonary phenotypes (Resistance, Compliance, Elastance, FVC, FEV0.1 and FEV0.1%) were evaluated in WT, Prss8+/-, C57/BL6J-βENaC-Tg and souble mutant Prss8+/- C57/BL6J-βENaC-Tg mice. n=3 mice/group. *p < 0.05, **p < 0.01, ***p < 0.001, versus WT mice; #p < 0.05, ###p < 0.001 versus C57/BL6J-βENaC-Tg mice; Student's t test. n.s. not significant.

Table. S1. Genes Increased in the lung tissue of ENaC-Tg mice (2-fold≤, Cy3≥20)

RefSeq_id	Enterz_id	symbol	description	Cy5 (WT)	Cy3 (ENaC-Tg)	Cy3/Cy5 (fold)
NM_178241	227288	Il8ra	interleukin 8 receptor, alpha	8.63	203.36	23.58
NM_025989	67133	Gp2	glycoprotein 2 (zymogen granule membrane)	4.73	74.91	15.83
NM_009141	100044702	Cxcl5	chemokine (C-X-C motif) ligand 5	10.22	133.71	13.08
NM_053113	93726	Ear11	eosinophil-associated, ribonuclease A family, member 11	5.13	66.09	12.88
NM_016960	20297	Ccl20	chemokine (C-C motif) ligand 20	10.75	127.40	11.85
NM_011315	20210	Saa3	serum amyloid A 3	58.11	652.21	11.22
XM_139051	214922	Slc39a2	solute carrier family 39 (zinc transporter), member 2	24.60	246.27	10.01
NM_008176	14825	Cxcl1	chemokine (C-X-C motif) ligand 1	19.67	182.34	9.27
NM_028946	74446	Slc9b1	RIKEN cDNA 4933425K02 gene	6.20	49.25	7.95
NM_010584	16429	Itlna	intelectin a	4.98	38.90	7.82
NM_053110	93695	Gpnmb	glycoprotein (transmembrane) nmb	175.11	1214.87	6.94
NM_206535	271375	Cd200r2	Cd200 receptor 2	8.32	57.10	6.86
NM_133245	51327	Ahsp	alpha hemoglobin stabilizing protein (Ahsp)	78.35	515.11	6.57
NM_010766	17167	Marco	macrophage receptor with collagenous structure	164.80	1060.77	6.44
-	668389	IGHV2-4	Immunoglobulin heavy chain V gene segment	12.01	74.99	6.25
NM_145126	104183	Chi3l4	chitinase 3-like 4	405.04	2506.70	6.19
NM_007802	13038	Ctsk	cathepsin K	171.24	1055.24	6.16
-	667859	IGKV8-18	Immunoglobulin Kappa light chain V gene segment	4.41	26.89	6.10
NM_020498	57248	Ly6i	lymphocyte antigen 6 complex, locus I	45.00	265.66	5.90
NM_017474	23844	Clca3	chloride channel calcium activated 3	6.90	40.25	5.83
NM_145594	234199	Fgl1	fibrinogen-like protein 1	4.18	23.88	5.71
XM_138376	238448	Ighv1-62-2	Immunoglobulin heavy chain V gene segment	30.20	165.25	5.47
NM_008046	14313	Fst	follistatin	13.49	73.68	5.46
NM_183249	66107	1100001G20Rik	RIKEN cDNA 1100001G20 gene	167.03	897.54	5.37
-	780799	IGHV7-1	Immunoglobulin heavy chain V gene segment	21.46	110.41	5.15
NM_008694	18054	Ngp	neutrophilic granule protein	8.77	45.09	5.14
XM_357633	620017	IGKV5-39	Immunoglobulin Kappa light chain V gene segment	48.60	247.10	5.08
NM_133862	99571	Fgg	fibrinogen, gamma polypeptide	16.64	83.81	5.04
NM_010479	193740	Hspa1a	heat shock protein 1A	198.37	978.64	4.93
NM_009892	12655	Chi3l3	chitinase 3-like 3	7566.47	36918.29	4.88
NM_009140	20311	Cxcl5	chemokine (C-X-C motif) ligand 5	11.88	57.36	4.83
NM_011867	23985	Slc26a4	solute carrier family 26, member 4	12.92	60.72	4.70
-	619833	IGHV1-69	Immunoglobulin heavy chain V gene segment	31.21	145.42	4.66
NM_001286493	546038	Spag11b	predicted gene, EG546038	33.02	153.78	4.66
XM_138377	382695	IGHV1-56	Immunoglobulin heavy chain V gene segment	131.01	606.10	4.63
NM_009921	12796	Camp	cathelicidin antimicrobial peptide	10.06	45.37	4.51
-	384411	IGKV12-89	Immunoglobulin Kappa light chain V gene segment	6.95	30.26	4.35
NM_007739	12837	Col8a1	procollagen, type VIII, alpha 1	13.14	54.98	4.18
XM_357683	384514	IGKV4-57-1	Immunoglobulin Kappa light chain V gene segment	148.30	618.50	4.17
NM_145603	234671	Ces2	carboxylesterase 2	5.04	20.81	4.13
NM_011337	20302	Ccl3	chemokine (C-C motif) ligand 3	51.54	209.59	4.07
NM_207281	403183	4832428D23Rik	RIKEN cDNA 4832428D23 gene	11.22	45.44	4.05
NM_007739	12837	COL8A1	collagen, type VIII, alpha 1	58.40	232.72	3.98
M34982	-	IGHV1S52	Immunoglobulin heavy chain V gene segment	126.99	503.93	3.97
XM_111360	28453	IGHV3-6	Immunoglobulin heavy chain V gene segment	64.83	254.06	3.92
NM_174998	170638	Hpcal4	hippocalcin-like 4	7.73	30.23	3.91
NM_031254	83433	Trem2	triggering receptor expressed on myeloid cells 2	71.80	277.93	3.87
XM_129391	71884	Chit1	chitinase 1 (chitotriosidase)	20.45	78.33	3.83
NM_009890	12642	Ch25h	cholesterol 25-hydroxylase	86.57	327.90	3.79
NM_145227	246728	Oas2	2'-5' oligoadenylate synthetase 2	17.47	65.10	3.73
XM_203589	640979	IGHV1S5	Immunoglobulin heavy chain V gene segment	71.73	266.36	3.71
NM_008920	19074	Prg2	proteoglycan 2, bone marrow	8.21	30.41	3.70
-	-	IGHV1S7	Immunoglobulin heavy chain V gene segment	101.05	372.95	3.69
NM_207244	271375	Cd200r2	Cd200 receptor 2	65.26	236.67	3.63
NM_133362	170942	Erdr1	rythroid differentiation regulator 1	847.02	3062.68	3.62
NM_009139	20305	Ccl6	chemokine (C-C motif) ligand 6	882.07	3166.88	3.59
XM_283962	74152	Stra6l	RIKEN cDNA 1300002K09 gene	7.38	26.37	3.57
NM_007739	12837	Col8a1	procollagen, type VIII, alpha 1	39.83	140.77	3.53
XM_355832	-	IGKV4-58	Immunoglobulin Kappa light chain V gene segment	53.35	187.89	3.52
NM_134160	171166	Mcoln3	mucolipin 3	38.25	134.68	3.52
-	-	IGHV1-55	Immunoglobulin Kappa light chain V gene segment	152.19	529.14	3.48
XM_356629	-	IGHV1-56	Immunoglobulin Kappa light chain V gene segment	47.55	163.18	3.43
NM_013532	14728	Lilrb4	leukocyte immunoglobulin-like receptor, subfamily B, member 4	177.68	590.79	3.32
XM_127186	-	IGHV1-11	Immunoglobulin heavy chain V gene segment	164.27	545.79	3.32
NM_177836	23792	Adam23	a disintegrin and metallopeptidase domain 23	7.46	24.63	3.30
XM_138324	-	IGHV6S4	Immunoglobulin heavy chain V gene segment	8.20	27.02	3.30
-	-	-	unknown	324.36	1056.43	3.26
-	-	-	unknown	277.82	901.17	3.24
NM_027552	70789	Kynu	kynureninase (L-kynurenine hydrolase)	21.85	70.26	3.22
NM_008605	17381	Mmp12	matrix metallopeptidase 12	32.21	102.88	3.19
NM_013532	14728	Lilrb4	leukocyte immunoglobulin-like receptor, subfamily B, member 4	253.48	807.33	3.18
NM_198164	78334	Cdc2l6	cell division cycle 2-like 6 (CDK8-like)	172.64	547.61	3.17
XM_487248	-	IGHV1S125	Immunoglobulin heavy chain V gene segment	193.91	614.75	3.17
NM_172814	239393	Lrp12	low density lipoprotein-related protein 12	87.12	275.37	3.16
NM_021364	23845	Clec5a	C-type lectin domain family 5, member a	15.90	50.06	3.15
NM_145222	227327	B3gnt7	UDP-GlcNAc:betaGal beta-1,3-N-acetylglucosaminyltransferase 7	60.91	187.42	3.08
NM_013655	20315	Cxcl12	chemokine (C-X-C motif) ligand 12	1194.92	3675.63	3.08
XM_487240	634100	IGHV1-31	Immunoglobulin heavy chain V gene segment	12.85	39.19	3.05

Table. S1. Genes Increased in the lung tissue of ENaC-Tg mice (2-fold≤, Cy3≥20)

RefSeq_id	Enterz_id	symbol	description	Cy5 (WT)	Cy3 (ENaC-Tg)	Cy3/Cy5 (fold)
NM_009969	12981	Csf2	colony stimulating factor 2 (granulocyte-macrophage)	23.02	69.99	3.04
NM_020509	57262	Retnla	resistin like alpha	841.60	2554.65	3.04
XM_138364	-	IGHV1S6	Immunoglobulin heavy chain V gene segment	89.15	269.52	3.02
NM_007388	11433	Acp5	acid phosphatase 5, tartrate resistant	174.80	527.97	3.02
NM_011819	23886	Gdf15	growth differentiation factor 15	41.00	123.06	3.00
NM_029931	70122	Mllt3	myeloid/lymphoid or mixed lineage-leukemia translocation to 3 homolog (Drosophila)	10.77	32.17	2.99
NM_008611	17394	Mmp8	matrix metallopeptidase 8	108.36	322.88	2.98
NM_001168304	78334	Cdk19	cyclin-dependent kinase 19	15.90	47.15	2.97
NM_021325	57781	Cd200r1	CD200 receptor 1	32.47	95.75	2.95
NM_008491	16819	Lcn2	lipocalin 2	1671.51	4922.58	2.94
010512;NM_184	16000	Igf1	insulin-like growth factor 1	90.28	263.33	2.92
NM_013559	-	Hsp110	heat shock protein 110	108.81	314.37	2.89
-	-	-	unknown	7.22	20.77	2.88
NM_018746	16427	Itih4	inter alpha-trypsin inhibitor, heavy chain 4	446.02	1280.97	2.87
XM_138378	636126	IGHV1S53	Immunoglobulin heavy chain V gene segment	69.27	198.40	2.86
NM_025844	66917	Chordc1	cysteine and histidine-rich domain (CHORD)-containing, zinc-binding protein 1	103.19	295.25	2.86
NM_021334	16411	Itgax	integrin alpha X	234.95	664.11	2.83
NM_013559	15505	Hsph1	predicted gene, ENSMUSG00000053750	30.14	84.55	2.80
NM_010934	18166	Npy1r	neuropeptide Y receptor Y1	26.03	72.78	2.80
NM_145515	226778	Mark1	MAP/microtubule affinity regulating kinase 1	51.07	142.36	2.79
NM_133775	77125	Il33	interleukin 33	732.03	2035.04	2.78
NM_011331	20293	Ccl12	chemokine (C-C motif) ligand 12	41.75	115.79	2.77
NM_019911	56720	Tdo2	tryptophan 2,3-dioxygenase	20.83	57.65	2.77
NM_010634	16592	Fabp5	fatty acid binding protein 5, epidermal	110.57	300.43	2.72
-	-	IGKV4-54	Immunoglobulin Kappa light chain V gene segment	48.96	132.32	2.70
-	-	IGKV4-72	Immunoglobulin Kappa light chain V gene segment	197.99	533.35	2.69
XM_135414	-	IGKV4-57	Immunoglobulin Kappa light chain V gene segment	62.65	167.72	2.68
XM_888439	623924	Gm6462	predicted gene 6462	238.41	632.52	2.65
NM_027836	109225	Ms4a7	membrane-spanning 4-domains, subfamily A, member 7	101.80	264.19	2.60
NM_009853	12514	Cd68	CD68 antigen	429.97	1100.12	2.56
-	-	IGKV4-91	Immunoglobulin Kappa light chain V gene segment	71.44	180.81	2.53
XM_131683	76574	Mfsd2	major facilitator superfamily domain containing 2	66.74	168.30	2.52
NM_145467	223272	Itgbl1	integrin, beta-like 1	65.44	164.62	2.52
NM_008768	18405	Orm1	orosomucoid 1	12.69	31.86	2.51
NM_023655	72169	Trim29	tripartite motif protein 29	9.05	22.57	2.50
NM_010104	13614	Edn1	endothelin 1	572.74	1427.75	2.49
NM_175406	242341	Atp6v0d2	ATPase, H+ transporting, lysosomal V0 subunit D2	435.61	1083.13	2.49
NM_008590	17294	Mest	mesoderm specific transcript	21.18	52.54	2.48
XM_111360	-	IGHV3-5	Immunoglobulin heavy chain V gene segment	15.68	38.85	2.48
NM_031198	21426	Tcfec	transcription factor EC	35.02	86.51	2.47
XM_111361	100045791	IGHV9-3	Immunoglobulin heavy chain V gene segment	36.09	89.02	2.47
NM_016737	20867	Stip1	stress-induced phosphoprotein 1	304.89	749.13	2.46
XM_356617	-	IGHV1S99	Immunoglobulin heavy chain V gene segment	149.99	367.62	2.45
NM_011031	18452	P4ha2	procollagen-proline, 2-oxoglutarate 4-dioxygenase (proline 4-hydroxylase), alpha II polypeptide	109.06	265.16	2.43
NM_010819	17474	Clec4d	C-type lectin domain family 4, member d	183.45	444.30	2.42
NM_007969	14038	Wfdc18	WAP four-disulfide core domain 18	12.08	29.11	2.41
NM_144943	246278	Cd207	CD 207 antigen	14.00	33.60	2.40
-	-	IGKV4-71	Immunoglobulin Kappa light chain V gene segment	150.94	361.66	2.40
NM_016812	53325	Banp	Btg3 associated nuclear protein	318.07	759.85	2.39
NM_001004174	433470	AA467197	expressed sequence AA467197	46.79	111.67	2.39
XM_134720	235130	Adamts15	a disintegrin-like and metallopeptidase (reprolysin type) with thrombospondin type 1 motif, 15	209.73	498.76	2.38
NM_172589	218454	Lhfpl2	lipoma HMGIC fusion partner-like 2	75.25	178.66	2.37
-	-	IGHV1-20	Immunoglobulin heavy chain V gene segment	278.87	661.36	2.37
NM_031999	83924	Gpr137b	G protein-coupled receptor 137B	196.67	465.96	2.37
-	-	IGKV4-55	Immunoglobulin Kappa light chain V gene segment	141.82	334.75	2.36
-	-	-	unknown	108.64	256.19	2.36
NM_011260	19695	Reg3g	regenerating islet-derived 3 gamma	337.37	792.49	2.35
XM_129166	77836	Mlana	melan-A	20.62	48.08	2.33
-	108857	Ankhd1	ankyrin repeat and KH domain containing 1	9.52	22.16	2.33
NM_009303	20972	Syngr1	synaptogyrin 1	132.86	309.26	2.33
NM_009983	13033	Ctsd	cathepsin D	5658.60	13154.38	2.32
NM_010188	14131	Fcgr3	Fc receptor, IgG, low affinity III	297.07	689.81	2.32
NM_144559	246256	Fcgr3a	Fc fragment of IgG, low affinity IIIa, receptor	106.69	247.35	2.32
NR_073011	381715	4930572O03Rik	RIKEN cDNA 4930572O03 gene	29.32	67.95	2.32
-	-	IGHV1S55	Immunoglobulin heavy chain V gene segment	180.68	417.02	2.31
NM_025759	360220	Speer4d	spermatogenesis associated glutamate (E)-rich protein 4d	23.23	53.59	2.31
NM_029796	76905	Lrg1	leucine-rich alpha-2-glycoprotein 1	876.17	2020.68	2.31
NM_001039562	654824	Ankrd37	ankyrin repeat domain 37	26.03	59.71	2.29
NM_008599	17329	Cxcl9	chemokine (C-X-C motif) ligand 9	15.97	36.60	2.29
NM_027988	71893	Noxo1	NADPH oxidase organizer 1	30.73	70.28	2.29
NM_008522	17002	Ltf	lactotransferrin	65.12	148.89	2.29
NM_011612	21942	Tnfrsf9	tumor necrosis factor receptor superfamily, member 9	10.54	23.81	2.26
NM_008083	14432	Gap43	growth associated protein 43	288.38	649.71	2.25
NM_010234	14281	Fos	FBJ osteosarcoma oncogene	387.03	870.57	2.25
NM_029103	74840	Armet	arginine-rich, mutated in early stage tumors	843.37	1894.12	2.25
NM_153101	235712	Mrgpra2	MAS-related GPR, member A2	11.67	26.17	2.24
NM_008298	100048597	Dnaja1	DnaJ (Hsp40) homolog, subfamily A, member 1	913.76	2039.89	2.23
NM_145144	108897	2810003C17Rik	RIKEN cDNA 2810003C17 gene	42.13	93.73	2.23

Table. S1. Genes Increased in the lung tissue of ENaC-Tg mice (2-fold≤, Cy3≥20)

RefSeq_id	Enterz_id	symbol	description	Cy5 (WT)	Cy3 (ENaC-Tg)	Cy3/Cy5 (fold)
NM_010442	15368	Hmox1	heme oxygenase (decycling) 1	232.62	514.46	2.21
NM_011146	19016	Pparg	peroxisome proliferator activated receptor gamma	112.58	248.45	2.21
NR_040420	625253	Dnajb9	DnaJ (Hsp40) homolog, subfamily B, member 9	125.21	276.27	2.21
XM_142364	100047132	IGKV6-20	Immunoglobulin Kappa light chain V gene segment	44.87	98.96	2.21
NM_013825	17076	Ly75	lymphocyte antigen 75	67.24	148.26	2.20
XM_138237	238393	Serpina3f	serine (or cysteine) peptidase inhibitor, clade A, member 3F	35.81	78.95	2.20
NM_007752	12870	Cp	ceruloplasminprovided	1156.31	2548.47	2.20
NM_183187	268709	Fam107a	cDNA sequence BC055107	9.83	21.65	2.20
NM_007469	11812	Apoc1	apolipoprotein C-I	320.78	706.74	2.20
NM_029639	76509	Plet1	RIKEN cDNA 1600029D21 gene	944.37	2071.43	2.19
-	-	IGKV16-104	Immunoglobulin Kappa light chain V gene segment	337.81	738.27	2.19
NM_013489	12523	Cd84	CD84 antigen	112.41	244.90	2.18
NM_009263	20750	Spp1	secreted phosphoprotein 1	974.98	2122.61	2.18
NM_007913	13653	Egr1	early growth response	466.30	1013.73	2.17
NM_011020	18415	Hspa4l	heat shock protein 4 like	132.69	287.63	2.17
NM_007408	11520	Adfp	adipose differentiation related protein	771.95	1665.59	2.16
NM_011539	21391	Tbxas1	thromboxane A synthase 1, platelet	127.89	275.39	2.15
NM_008620	76074	Gbp8	cDNA sequence BC057170	117.61	252.98	2.15
NM_010762	17153	Mal	myelin and lymphocyte protein, T-cell differentiation protein	269.36	579.20	2.15
NM_145953	107869	Cth	cystathionase (cystathionine gamma-lyase)	41.12	88.40	2.15
NM_010172	14068	F7	coagulation factor VII	102.04	217.17	2.13
NM_022430	64381	Ms4a8a	membrane-spanning 4-domains, subfamily A, member 8A	212.00	449.74	2.12
NM_024406	11770	Fabp4	fatty acid binding protein 4, adipocyte	453.38	960.71	2.12
NM_009251	20715	Serpina3g	serine (or cysteine) peptidase inhibitor, clade A, member 3G	63.60	134.50	2.11
NM_026931	69068	1810011O10Rik	RIKEN cDNA 1810011O10 gene	399.48	844.01	2.11
XM_485781	-	IGKV5-48	Immunoglobulin Kappa light chain V gene segment	59.80	126.28	2.11
NM_009779	12267	C3ar1	complement component 3a receptor 1	27.48	57.97	2.11
NM_011780	23792	Adam23	a disintegrin and metallopeptidase domain 23	14.44	30.44	2.11
-	791346	Gm10118	predicted gene, ENSMUSG00000062561	141.45	297.62	2.10
NM_011414	20568	Slpi	secretory leukocyte peptidase inhibitor	460.54	968.53	2.10
NM_144520	67815	Sec14l2	SEC14-like 2 (S. cerevisiae)	209.53	438.33	2.09
-	675749	Gm10693	predicted pseudogene 10693	37.19	77.70	2.09
NM_028808	74191	P2ry13	purinergic receptor P2Y, G-protein coupled 13 [Source:MarkerSymbol;Acc:MGI:1921441]	42.63	88.95	2.09
NM_008147	14727	Gp49a	leukocyte immunoglobulin-like receptor, subfamily B, member 4B	118.97	247.46	2.08
NM_013650	20201	S100a8	S100 calcium binding protein A8 (calgranulin A)	3273.25	6803.14	2.08
NM_008198	14962	Cfb	complement factor B	423.72	879.13	2.07
NM_145434	217166	Nr1d1	nuclear receptor subfamily 1, group D, member 1	32.52	67.03	2.06
NM_172542	77739	Adamtsl1	ADAMTS-like 1	20.41	42.01	2.06
NM_010588	16450	Jag2	jagged 2	28.74	59.15	2.06
NM_008223	15160	Serpind1	serine (or cysteine) peptidase inhibitor, clade D, member 1	14.48	29.78	2.06
NM_013560	15507	Hspb1	heat shock protein 1	2177.34	4477.24	2.06
NM_024495	71934	Car13	carbonic anhydrase 13	12.43	25.54	2.05
022431;NM_026	68774	Ms4a6d	membrane-spanning 4-domains, subfamily A, member 6D	196.16	402.93	2.05
NM_011333	20296	Ccl2	chemokine (C-C motif) ligand 2	49.33	101.25	2.05
NM_031165	15481	Hspa8	heat shock protein 8	3990.34	8174.50	2.05
NM_011407	20555	Ap2b1	adaptor-related protein complex 2, beta 1 subunit	33.92	69.46	2.05
NM_009786	12301	Cacybp	calcyclin binding protein	1466.62	3002.36	2.05
-	-	IGKV19-93	Immunoglobulin Kappa light chain V gene segment	158.38	321.86	2.03
NM_172679	229227	4932438A13Rik	RIKEN cDNA 4932438A13 gene	59.01	119.87	2.03
NM_007444	11702	Amd1	S-adenosylmethionine decarboxylase 1	110.90	224.82	2.03
NM_016704	12274	C6	complement component 6	189.96	384.12	2.02
NM_177084	110895	Slc9a4	solute carrier family 9 (sodium/hydrogen exchanger), member 4	10.17	20.51	2.02
NM_024435	67405	Nts	neurotensin	11.97	24.10	2.01
NM_007675	26366	Ceacam10	carcinoembryonic antigen-related cell adhesion molecule 10	18.97	38.17	2.01
NM_010554	16175	Il1a	interleukin 1 alpha	18.41	37.00	2.01
NM_009912	12768	Ccr1	chemokine (C-C motif) receptor 1	24.01	48.17	2.01
NM_008303	628438	Hspe1-rs1	heat shock protein 1 (chaperonin 10), related sequence 1	1125.36	2257.46	2.01
NM_011124	100042493	Ccl21b	chemokine (C-C motif) ligand 21b	2142.49	4290.31	2.00

Table. S2. Genes Decreased in the lung tissue of ENaC-Tg mice (0.5-fold≥, Cy5≥20)

RefSeq_id	Enterz_id	symbol	description	Cy5 (WT)	Cy3 (ENaC-Tg)	Cy3/Cy5 (fold)
NM_016771	53315	Sult1d1	sulfotransferase family 1D, member 1	78.64	11.86	0.15
NM_198028	241197	Serpinb10	serine (or cysteine) peptidase inhibitor, clade B (ovalbumin), member 10	60.06	12.38	0.21
NM_011681	22287	Scgb1a1	secretoglobin, family 1A, member 1 (uteroglobin)	65031.42	14646.36	0.23
NM_023612	71690	Esm1	endothelial cell-specific molecule 1	314.03	70.78	0.23
NM_010444	15370	Nr4a1	nuclear receptor subfamily 4, group A, member 1	1038.59	239.47	0.23
NM_011126	18843	Plunc	palate, lung, and nasal epithelium carcinoma associated	676.45	179.30	0.27
NM_008343	16009	Igfbp3	insulin-like growth factor binding protein 3	327.73	92.32	0.28
NM_001278161	15129	Hbb-b1	hemoglobin, beta adult major chain	62517.42	18607.36	0.30
NM_009695	11813	Apoc2	apolipoprotein C-II	117.04	38.80	0.33
NM_175012	225642	Grp	gastrin releasing peptide	82.67	28.09	0.34
NM_008218	110257	Hba-a1	hemoglobin alpha, adult chain 1	57378.42	20249.72	0.35
NM_007621	12409	Cbr2	carbonyl reductase 2	38360.42	13576.28	0.35
XM_144122	242805	Ankrd65	ankyrin repeat domain 65	36.87	13.24	0.36
-	-	-	unknown	35.73	12.83	0.36
NM_009714	11889	Asgr1	asialoglycoprotein receptor 1	46.34	16.74	0.36
NM_011784	23796	Agtrl1	angiotensin receptor-like 1	454.59	165.64	0.36
-	-	-	unknown	34.77	12.69	0.36
NM_173052	282663	Serpinb1b	serine (or cysteine) peptidase inhibitor, clade B, member 1b	22.68	8.28	0.37
XM_110691	75863	Clec4g	C-type lectin domain family 4, member g	25.61	9.70	0.38
XM_287054	68874	1190002J23Rik	RIKEN cDNA 1190002J23 gene	32.18	12.25	0.38
NM_028848	74717	Spata17	spermatogenesis associated 17	24.18	9.45	0.39
NM_008311	15559	Htr2b	5-hydroxytryptamine (serotonin) receptor 2B	27.92	11.29	0.40
XM_133956	213002	Ifitm6	interferon induced transmembrane protein 6	289.45	118.27	0.41
XM_129390	69169	Faim3	Fas apoptotic inhibitory molecule 3	506.40	208.34	0.41
NM_019487	56016	Hebp2	heme binding protein 2	23.47	9.69	0.41
NM_054038	117158	Scgb3a2	secretoglobin, family 3A, member 2	20687.05	8603.05	0.42
NM_009234	20666	Sox11	SRY (sex determining region Y)-box 11	79.25	32.99	0.42
NM_009922	12797	Cnn1	calponin 1	595.95	248.24	0.42
NM_007817	13107	Cyp2f2	cytochrome P450, family 2, subfamily f, polypeptide 2	16658.78	6961.71	0.42
XM_131840	230899	Nppa	natriuretic peptide precursor type A	95.54	40.39	0.42
NM_013613	18227	Nr4a2	nuclear receptor subfamily 4, group A, member 2	147.89	62.91	0.43
XM_128291	68311	Lypd2	Ly6/Plaur domain containing 2	508.95	224.07	0.44
NM_001033170	73813	Fam83e	RIKEN cDNA 4930403C10 gene	27.43	12.09	0.44
NM_009749	12069	Bex2	brain expressed X-linked 2	654.07	290.15	0.44
NM_007719	12775	Ccr7	chemokine (C-C motif) receptor 7	166.16	73.72	0.44
NM_023597	71354	Wdr31	WD repeat domain 31	57.65	25.73	0.45
NM_011620	21957	Tnnt3	troponin T3, skeletal, fast	35.65	15.95	0.45
XM_134806	70989	4931429I11Rik	RIKEN cDNA 4931429I11 gene	25.07	11.28	0.45
NM_029064	74711	Ttll9	tubulin tyrosine ligase-like family, member 9	25.84	11.66	0.45
NM_029627	76486	Ly6k	lymphocyte antigen 6 complex, locus K	35.48	16.05	0.45
NM_017372	17105	Lyzs	lysozyme	56345.42	25566.51	0.45
NM_021278	100047211	Tmsb4x	thymosin, beta 4, X chromosome	49135.42	22309.22	0.45
NM_001081977	108089	Rnf144	ring finger protein 144 [Source:MarkerSymbol;Acc:MGI:1344401]	20.25	9.25	0.46
NM_177876	330192	Vps37b	vacuolar protein sorting 37B (yeast)	22.50	10.35	0.46
NM_183094	667387	Xlr4b	X-linked lymphocyte-regulated 4B	26.62	12.55	0.47
XM_485890	72383	Cnfn	cornifelin	142.03	67.15	0.47
NM_011580	640441	Thbs1	thrombospondin 1	67.46	31.93	0.47
XM_489730	71236	Rtdr1	rhabdoid tumor deletion region gene 1	39.11	18.55	0.47
NM_007641	12482	Ms4a1	membrane-spanning 4-domains, subfamily A, member 1	561.05	268.25	0.48
NM_172050	217306	Cd300e	CD300e antigen	61.22	29.40	0.48
NM_008182	14858	Gsta2	glutathione S-transferase, alpha 2 (Yc2)	81.64	39.36	0.48
NM_153391	213081	Wdr19	WD repeat domain 19	51.80	25.13	0.49
NM_138949	192651	Zfp286	zinc finger protein 286 [Source:MarkerSymbol;Acc:MGI:2384758]	27.74	13.60	0.49
NM_009189	20471	Six1	sine oculis-related homeobox 1 homolog (Drosophila)	37.64	18.53	0.49
NM_138652	192113	Atp12a	ATPase, H+/K+ transporting, nongastric, alpha polypeptide	21.97	10.83	0.49
NM_007549	12143	Blk	B lymphoid kinase	46.11	22.82	0.49
-	-	-	unknown	25.90	12.84	0.50
NM_007812	-	Cyp2a5	cytochrome P450, family 2, subfamily a, polypeptide 5	116.43	57.76	0.50
